# Automatic autism spectrum disorder detection using artificial intelligence methods with MRI neuroimaging: A review

**DOI:** 10.3389/fnmol.2022.999605

**Published:** 2022-10-04

**Authors:** Parisa Moridian, Navid Ghassemi, Mahboobeh Jafari, Salam Salloum-Asfar, Delaram Sadeghi, Marjane Khodatars, Afshin Shoeibi, Abbas Khosravi, Sai Ho Ling, Abdulhamit Subasi, Roohallah Alizadehsani, Juan M. Gorriz, Sara A. Abdulla, U. Rajendra Acharya

**Affiliations:** ^1^Faculty of Engineering, Science and Research Branch, Islamic Azad University, Tehran, Iran; ^2^Department of Computer Engineering, Ferdowsi University of Mashhad, Mashhad, Iran; ^3^Faculty of Electrical and Computer Engineering, Semnan University, Semnan, Iran; ^4^Neurological Disorders Research Center, Qatar Biomedical Research Institute, Hamad Bin Khalifa University, Qatar Foundation, Doha, Qatar; ^5^Department of Medical Engineering, Mashhad Branch, Islamic Azad University, Mashhad, Iran; ^6^Data Science and Computational Intelligence Institute, University of Granada, Granada, Spain; ^7^Institute for Intelligent Systems Research and Innovation (IISRI), Deakin University, Geelong, VIC, Australia; ^8^Faculty of Engineering and IT, University of Technology Sydney (UTS), Ultimo, NSW, Australia; ^9^Faculty of Medicine, Institute of Biomedicine, University of Turku, Turku, Finland; ^10^Department of Computer Science, College of Engineering, Effat University, Jeddah, Saudi Arabia; ^11^Ngee Ann Polytechnic, Singapore, Singapore; ^12^Department of Biomedical Informatics and Medical Engineering, Asia University, Taichung, Taiwan; ^13^Department of Biomedical Engineering, School of Science and Technology, Singapore University of Social Sciences, Singapore, Singapore

**Keywords:** ASD diagnosis, neuroimaging, MRI modalities, machine learning, deep learning

## Abstract

Autism spectrum disorder (ASD) is a brain condition characterized by diverse signs and symptoms that appear in early childhood. ASD is also associated with communication deficits and repetitive behavior in affected individuals. Various ASD detection methods have been developed, including neuroimaging modalities and psychological tests. Among these methods, magnetic resonance imaging (MRI) imaging modalities are of paramount importance to physicians. Clinicians rely on MRI modalities to diagnose ASD accurately. The MRI modalities are non-invasive methods that include functional (fMRI) and structural (sMRI) neuroimaging methods. However, diagnosing ASD with fMRI and sMRI for specialists is often laborious and time-consuming; therefore, several computer-aided design systems (CADS) based on artificial intelligence (AI) have been developed to assist specialist physicians. Conventional machine learning (ML) and deep learning (DL) are the most popular schemes of AI used for diagnosing ASD. This study aims to review the automated detection of ASD using AI. We review several CADS that have been developed using ML techniques for the automated diagnosis of ASD using MRI modalities. There has been very limited work on the use of DL techniques to develop automated diagnostic models for ASD. A summary of the studies developed using DL is provided in the Supplementary Appendix. Then, the challenges encountered during the automated diagnosis of ASD using MRI and AI techniques are described in detail. Additionally, a graphical comparison of studies using ML and DL to diagnose ASD automatically is discussed. We suggest future approaches to detecting ASDs using AI techniques and MRI neuroimaging.

## Introduction

A complex intricate network of millions of neurons is responsible for monitoring and controlling each part of the human body and brain (Sparks et al., 2002; Brieber et al., 2007; Ecker et al., 2015). These networks consist of many neurons that need to be directly interconnected and synchronized (Sato et al., 2012; Hernandez et al., 2015). It has been suggested that certain disorders in the human body arise when brain networks are incorrectly connected to manage a specific activity (Gautam and Sharma, 2020; Noor et al., 2020; Khodatars et al., 2021; Loh et al., 2022). Disorders of this type can be classified into three groups based on their psychological or neural characteristics and threaten the health of many individuals across the globe. Autism spectrum disorder (ASD) (Yang et al., 2022), schizophrenia (Sadeghi et al., 2022), attention deficit hyperactivity disorder (ADHD) (Bakhtyari and Mirzaei, 2022), epilepsy (Shoeibi et al., 2021a), Parkinson’s disease (Sahu et al., 2022), and bipolar disorder (BD) (Highland and Zhou, 2022) are some of the most known neurodevelopmental disorders.

Autism spectrum disorder is a neurodevelopmental disorder that manifests in childhood and causes a variety of challenges to individuals (Ecker et al., 2015). Those with ASD have difficulties with verbal and non-verbal communication, cognitive skills, social behavior, and entertaining activities (Aghdam et al., 2019; Ahmed et al., 2020a,b). ASD begins in the early stages of embryonic development, according to research results. Autism is thought to be caused by specific signal patterns in the cortex, abnormalities in the immune system, growth hormone fluctuations, and abnormalities in the neural mirror system in the embryonic stage (Chen et al., 2022; Jayanthy and Din, 2022). The overall ASD prevalence is one in 44 children aged 8 years, and ASD is around 4 times as prevalent among boys as among girls (Rakić et al., 2020; Maenner et al., 2021). In addition to lifelong social and adaptive disorders, one of the major consequences of autism is its negative impact on quality of life (Choi, 2017; Brown et al., 2018; Bengs et al., 2020; Byeon et al., 2020; D’Souza et al., 2020; Cao et al., 2021; Chen Y. et al., 2021; Chen H. et al., 2021; Chu et al., 2022). Therefore, early diagnosis and treatment of ASD are paramount for improving the quality of life of ASD children and their families (Kasari and Smith, 2013).

According to the DSM-3, mental health professionals originally divided autism into five categories, including Asperger’s syndrome, Rett syndrome, childhood disintegrative disorder (CDD), autistic disorder, and Pervasive developmental disorder-not otherwise specified (PDD-NOS) (Volkmar et al., 1992; Matson et al., 2009). Using this method, physicians observed the symptoms of autistic individuals and compared their observations to those in the DSM-3 to diagnose the specific type of autism (Volkmar et al., 1986, 1992; Matson et al., 2009). In 2013, the DSM-5 was published, making significant changes to the categorization of autism (Volkmar and Mcpartland, 2014). DSM-5 categorizes autism severity into three levels, and more information is given in Volkmar and Mcpartland (2014). According to DSM-5, the lower the severity level of autism, the less support the child requires. Autism individuals with the second and third severity levels show moderate to severe symptoms and therefore require more frequent support. Although the DSM-5 provides explanations of the autism spectrum, these explanations are incomplete and do not provide guidance on the specific support that autistic children may require. In addition, some individuals simply do not fall into any of these categories, and ASD can change and intensify over time (Kim et al., 2014; Volkmar and Mcpartland, 2014).

Early diagnosis of ASD is of utmost importance for specialist physicians (Akhavan Aghdam et al., 2018; Anirudh and Thiagarajan, 2019; Arya et al., 2020; Al-Hiyali et al., 2021; Almuqhim and Saeed, 2021; Bayram et al., 2021). Hereafter, clinical screening methods for diagnosing ASD are introduced, including autism diagnostic interview-revised (ADI-R), childhood autism rating scale (CARS), social responsiveness scale, autism diagnostic observation schedule (ADOS), and Joseph picture self-concept scale (Thabtah and Peebles, 2019). Clinical screening methods have been proven effective in diagnosing ASD and are of great interest to specialist physicians. Additionally, these methods assist in treating and preventing the development of ASD in the early stages (Thabtah and Peebles, 2019). As well as their many advantages, the mentioned methods always pose challenges for specialists (Thabtah and Peebles, 2019). These procedures involve long questionnaires, so they are very time-consuming and require different specialist physicians to analyze the questionnaire, which has led to many criticisms of clinical screening methods.

Additionally, some ASD diagnosis tools have been provided by neurologists and psychologists, including autism spectrum quotient (AQ), a modified checklist for autism in toddlers (M-CHAT), and a childhood Asperger syndrome test (CAST) (Thabtah and Peebles, 2019). Various items in these tools can be used to diagnose different types of autism; however, these methods face different challenges in the diagnosis of ASD (Thabtah and Peebles, 2019). These tools, for example, are not considered definitive screening methods for diagnosing ASD. Because, in most cases, ASD is diagnosed by them without the presence of a specialist physician (Thabtah and Peebles, 2019). However, some of these methods do not meet DSM-5 requirements (Thabtah and Peebles, 2019). Due to this, it is necessary to provide tools that are compatible with DSM-5.

Neuroimaging techniques are one group of methods used for diagnosing neurological and mental disorders such as ASD. These methods comprise structural and functional neuroimaging modalities, which are of special interest to physicians, particularly in diagnosing various brain disorders (Shoeibi et al., 2021b,2022c). The fMRI is one of the major functional neuroimaging methods that records data in a non-invasive manner. fMRI has a high spatial resolution, making it an excellent method for examining functional connectivity in the brain. fMRI data is classified into two categories: T-fMRI and rs-fMRI. Furthermore, fMRI data are composed of a 4-dimensional tensor, which permits the 3D volume of the brain to be segmented into smaller areas, and the activity of each area is recorded for a predetermined time period. Although fMRI has provided satisfactory results in diagnosing a variety of brain disorders, these techniques are costly and too sensitive to motion artifacts (Ghassemi et al., 2020; Shoeibi et al., 2022b).

Structural and DTI have been used to examine brain anatomy and the interaction between brain regions, respectively. The structural neuroimaging modalities offer the advantage of cost-effectiveness and the availability of imaging protocols in most treatment facilities (Ghassemi et al., 2020). Physicians use sMRI modalities to diagnose autism in autistic individuals using (i) geometric features and (ii) volumetric features, which physicians have used to demonstrate that autistic people demonstrate superior brain development in comparison to normal people (Brambilla et al., 2003; Siewertsen et al., 2015; Zürcher et al., 2015; Zhang and Roeyers, 2019). Hazlett et al. (2005) studied the brain structure of 51 autistic children and 25 normal children (1.5–3 years of age). Their findings indicated that the Cerebellum white matter volume of autistic children was 2–4 times greater than that of normal children.

Although MRIs offer many advantages, MRI artifacts reduce the accuracy with which clinicians are able to diagnose autism. Additionally, ASD individuals’ MRI data is recorded with multiple slices and different protocols. Consequently, it takes considerable time to examine all MRI slices accurately, and clinicians should be highly precise. The fatigue of the physician may lead to an incorrect diagnosis of ASD in many cases. In addition, MRI data is problematic because most physicians are inexperienced in interpreting these images and may find diagnosing ASD in its early stages difficult.

Numerous treatment methods have also been provided for ASD patients so far, some of which are listed here. Transcranial magnetic stimulation (TMS) and transcranial direct current stimulation (tDCS) are two non-invasive methods to diagnose and treat various neurological and mental disorders such as ASD (Khodatars et al., 2021). Using them, the areas of the brain where ASD occurs are selected by specialist physicians. Electrical pulses are then applied to these areas to treat or control ASD (Khodatars et al., 2021). Additionally, some researchers have provided rehabilitation systems based on AI techniques to treat ASD. For example, Cai et al. (2013) presented a virtual reality (VR) system for treating ASD. They proposed a VR program for people with ASD to interact with dolphins in their work. This tool enables people with ASD to virtually be at the pool as dolphin trainers, aiming to help people with ASD learn different types of non-verbal communication through hand movements with virtual dolphins.

To improve the accuracy of ASD diagnosis, AI techniques can be used. The use of AI in diagnosing various diseases has been studied (Nogay and Adeli, 2020; Ahmadi-Dastgerdi et al., 2021; Shoeibi et al., 2022a). Several studies have demonstrated that AI techniques, along with MRI neuroimaging modalities, increase the accuracy of ASD diagnosis (Nogay and Adeli, 2020; Ahmadi-Dastgerdi et al., 2021). An increasing number of studies have been conducted on detecting ASD using ML and DL methods. Researchers first demonstrated that ASD could be diagnosed from ML using MRI neuroimaging technologies (Shoeibi et al., 2022a). Based on ML algorithms, feature extraction, dimension reduction, and classification algorithms in CADS are selected through trial and error (Parikh et al., 2019; Alizadehsani et al., 2021). Choosing an appropriate algorithm for each CADS section can be challenging without adequate knowledge of AI (Mohammadpoor et al., 2016; Parikh et al., 2019; Alizadehsani et al., 2021; Wang et al., 2021a,c). Furthermore, ML techniques are not suitable for small data sets (Ghassemi et al., 2021). Therefore, these methods do not contribute to developing software for detecting ASDs using MRI neuroimaging modalities.

Various studies are being conducted to diagnose various diseases and disorders by using these methods to overcome the challenges inherent in ML techniques (Noor et al., 2019; Al-Shoukry et al., 2020; Altinkaya et al., 2020; Yao et al., 2020). For example, in contrast to ML methods, DL uses deep layers for feature extraction and classification and requires fewer implementation steps in diagnosing ASD (Goodfellow et al., 2016). Furthermore, DL-based CADS can be more efficient and accurate with large input data.

An overview of studies relating to the detection of ASD using MRI neuroimaging methods is presented in this comprehensive systematic review. The first step was to systematically review all publications on ASD detection using MRI modalities and ML techniques. A recent review by the authors of this review discussed the use of different neuroimaging modalities and DL architectures to detect ASD (Khodatars et al., 2021). Supplementary Appendix A presents a review paper describing ASD detection in different neuroimaging modalities using DL techniques to compare ML and DL studies.

The following sections describe the following. Section 2 is a search Strategy based on PRISMA guidelines. Section 3 reviews the review papers in AI techniques for ASD diagnosis. Section 4 describes the CADS based on AI to detect ASD from MRI neuroimaging images. Section 5 presents a comparison between ML and DL studies to ASD detection using MRI modalities. Section 6 examines the most critical challenges for detecting ASD using AI methods. Future directions and conclusion sections are presented in sections 7 and 8, respectively.

## Search strategy based on PRISMA guideline

The PRISMA protocol was used to select and review papers in this study (Sadeghi et al., 2022). Papers on the diagnosis of ASD by MRI modalities and AI models (ML and DL) published from 2016 to 2022 were included in this study. In this review paper, various citation databases, including IEEE, Wiley, Frontiers, ScienceDirect, SpringerLink, ACM, and ArXiv were used to search for papers in the field of ASD detection. Furthermore, Google Scholar has been used to search for the article entirety. Here are the keywords, including “ASD classification,” “Feature extraction,” “fMRI,” “sMRI,” and “Autism Spectrum Disorder,” which were used to search for articles relating to the diagnosis of ASD using ML algorithms. To search for articles related to DL, the keywords used were “Autism Spectrum Disorder,” “ASD,” fMRI,” “sMRI,” and “Deep Learning.”

**FIGURE 1:**
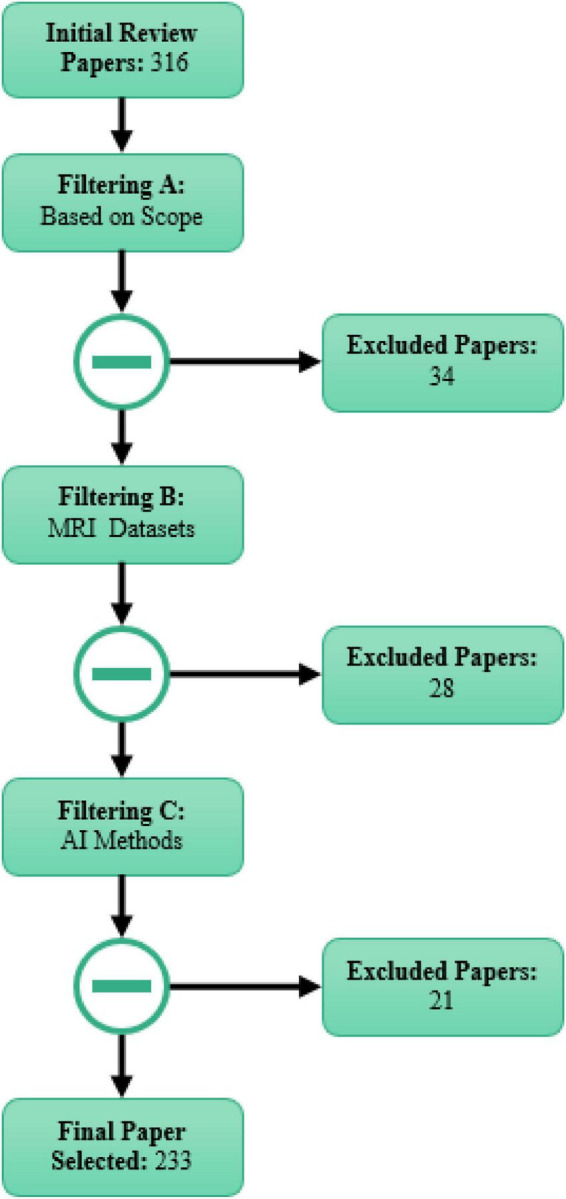
Papers selection process based on the PRISMA guidelines.

As stated above, papers were selected and reviewed based on the PRISMA protocol at three different levels. In the first level, 34 out of 316 downloaded papers were eliminated as they were out of the scope of this study. Then, 28 papers were also excluded as they did not use MRI datasets in the ASD diagnosis, followed by excluding further 21 papers due to no use of AI techniques. Therefore, 233 papers were finally selected and used in this review paper. Figure 1 shows the selection procedure of papers based on the PRISMA protocol on three levels. The key criteria for the inclusion and exclusion of papers on the ASD diagnosis based on the PRISMA protocol are shown in Table 1.

**TABLE 1:** The exclusion and inclusion criteria for diagnosis of ASD.

Inclusion	Exclusion
1. sMRI neuroimaging modalities	1. Treatment of ASD
2. fMRI neuroimaging modalities	2. Clinical methods for ASD treatment
3. Different Types of Autism	3. Rehabilitation systems for ASD detection (Without AI techniques)
4. DL models	
5. Feature extraction methods	
6. Dimension reduction methods	
7. Classification methods	

## Artificial intelligence techniques for autism spectrum disorder diagnosis

For researchers in cognitive sciences, autism is a well-recognized neurodevelopmental disorder with a high prevalence in recent years. Challenges in the ASD diagnosis for physicians have resulted in extensive studies on this brain disorder. Scholars in AI, and cognitive sciences seek to develop a real diagnostic tool for ASD using various AI techniques. Accordingly, extensive studies have focused on ASD diagnosis using neuroimaging modalities and AI techniques, outlined in this section by reviewing articles in the field of ASD diagnosis using these techniques.

Pagnozzi et al. (2018) reviewed 123 articles on ASD diagnosis using sMRI modalities and reported further developments in some brain areas of ASD individuals than those of HC. They also explained that ASD caused changes in the structure of patients’ brains, including increased volume of frontal and temporal lobes, increased thickness of the frontal cortex, and increased cerebrospinal fluid volume. This study assists scholars in applying AI techniques in ASD diagnosis from sMRI modalities in future studies.

Nogay and Adeli (2020) published a review article on ASD diagnosis using brain imaging and ML techniques. They reviewed studies on ASD diagnosis for sMRI, fMRI and combined data using ML techniques and found a higher accuracy of ASD diagnosis at younger ages. They hope to develop a practical ASD diagnostic tool based on ML techniques from MRI modalities.

In another study, Xu et al. (Shoeibi et al., 2022a) reported methods and tools associated with ASD diagnosis from MRI data based on ML techniques. Initially, they introduced the most important ML-based algorithms, including feature extraction, feature selection and reduction, training and test models, and evaluation parameters.

Parlett-Pellerit et al. (Ahmadi-Dastgerdi et al., 2021) reviewed studies on unsupervised ML techniques for ASD diagnosis. In this study, various clinical data and medical imaging data were discussed for ASD diagnosis using unsupervised ML techniques.

The most important feature selection and classification algorithms for ASD diagnosis were studied in Rahman et al. (2020) paper. Their input data comprises various psychological tests such as ADOS and MRI modalities. They claimed that this study could assist scholars in developing future studies on ADS diagnosis.

A review article on the diagnosis of ASD and ADHD using AI techniques was published by Eslami et al. (2021a). They discussed DL and ML-based studies on ASD and ADHD diagnosis from MRI modalities and the most important AI techniques (DL and ML). In the ML section, the authors presented the most important feature extraction techniques, such as effective dynamic connectivity, and outlined various popular DL techniques.

Khodatars et al. (2021) presented a review paper on ASD diagnosis and rehabilitation using DL techniques. They initially introduced the public neuroimaging modalities datasets, such as MRI, pre-processing techniques, and DL models, an ASD diagnosis. Then, they summarized the studies conducted in this field in a table. They also discussed studies in the field of autism rehabilitation using DL techniques.

In this section, the most important review papers on ASD diagnosis from various data and AI techniques were discussed. In our study, ASD diagnosis papers using MRI data and various AI techniques (ML and DL) were reviewed. This paper reports all ASD diagnosis articles from 2010 to 2022. Also, the most important challenges and future works for diagnosing ASD from MRI modalities are presented. To the best of our knowledge, no similar review article has been provided, and our review article has outstanding innovations.

## Computer-aided design systems for aided design systems diagnosis by magnetic resonance imaging neuroimaging modalities

**FIGURE 2:**
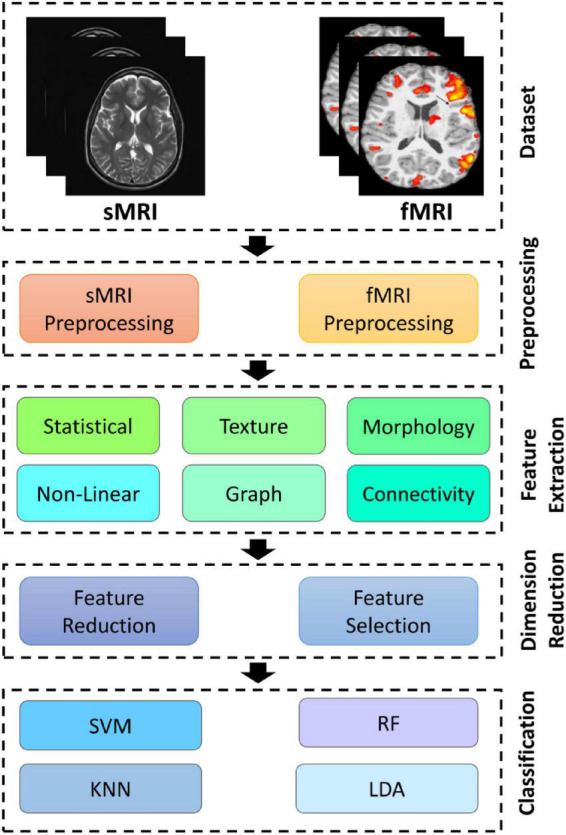
Block diagram of CADS- based on ML techniques for automated ASD diagnosis.

The application of CADS based on AI techniques is presented in this section and illustrated in Figure 2. The steps involved in CADS using ML for ASD detection are outlined in Figure 2. As shown in Figure 2, CADS input consists of datasets containing MRI modalities. Standard preprocessing (low-level) methods for MRI neuroimaging modalities were demonstrated as a second step. Next, we will discuss these preprocessing methods for MRI neuroimaging modalities. The third step involves feature extraction. Feature reduction or selection techniques (dimension reduction) are considered to be the fourth step in the CADS based on ML. The final step involves the use of classification algorithms. The only difference between DL-based and ML-based CADS is the feature extraction to the classification step. This procedure is carried out in deep layers in CADS based on DL. This enables the extraction of features from MRI data without the user’s intervention. Moreover, in CADS based on DL, diagnostics of ASD may be possible in case there are more input data, allowing the development of actual software for the detection of ASD. The details of ASD detection from MRI neuroimaging modalities using DL architectures are given in Supplementary Appendix (A). Here we present the details of CADS based on ML and some of the most important algorithms in each section.

### Magnetic resonance imaging neuroimaging autism spectrum disorder datasets

Various MRI modalities datasets for ASD diagnosis are available to researchers, including UCI (Last Access 19/07/2022a), NDAR (Last access 19/07/2022b), AGRE (Last access 19/07/2022d), NIMH Repository and Genomics Resource, n.d. (Last access 19/07/2022f), Gene Expression Omnibus [GEO], n.d. (Last access 19/07/2022e), SSC (Last access 19/07/2022h), Simons VIP SFARI (Last access 19/07/2022g), and autism brain imaging data exchange (ABIDE/) (Khodatars et al., 2021). Table 2 and Supplementary Table 1 summarize studies of ASD diagnosis using ML and DL techniques. As can be seen, the ABIDE database has a special place in research. ABIDE is recognized as the most complete and freely available database of MRI modalities for the automatic diagnosis of ASD (Khodatars et al., 2021). This dataset has two parts, ABIDE 1 and ABIDE-II, containing sMRI data, rs-fMRI data, and phenotypic data. 1112 datasets are involved in ABIDE I, and 1114 datasets are included in ABIDE II (Khodatars et al., 2021). ABIDE 1 also contains preprocessed data from MRI modalities for research, known as the preprocessed connectomes project (PCP) (Khodatars et al., 2021). Additionally, other available datasets, such as NDAR, UCI, and NRGR, have been used in ASD diagnostic. The results show that these datasets have been able to achieve satisfactory results. The datasets used for each study are summarized in Table 2 and Supplementary Table 1.

**TABLE 2:** Automated diagnosis of ASD with MRI neuroimaging modalities using ML methods.

References	Dataset	Number of cases	Modalities	Atlas + Pipeline	Feature extraction	Feature selection	Classification	The best performance criteria (%)
Haweel et al., 2020	NDAR	39 ASD	rs-fMRI	Brainnetome (BNT) Atlas	GLM Features	RFE	RF	Acc = 72
			sMRI	MNI-152 Atlas				
Yang X. et al., 2019	ABIDE	505 ASD, 530 HC	rs-fMRI	CC400 Atlas + CPAC Pipeline	Different Features	Nilearn	Ridge	Acc = 71.98 Pre = 71.53 Rec = 70.89
Zhang X. et al., 2020	NDAR	30 ASD, 30 HC	sMRI	NA	Cortical Path Signature Features	−	Siamese Verification Model	Acc = 87 Sen = 83 Spe = 90
Bi et al., 2019	ABIDE	103 ASD, 106 HC	rs-fMRI	AAL Atlas + DPARSF Pipeline	Graph-Theoretic Indicators (Dimensional Features)	−	GERSVMC	Acc = 96.8
Sartipi et al., 2018	ABIDE	222 ASD, 246 HC	rs-fMRI	HO Atlas + CPAC Pipeline	GARCH Model	*T*-test	SVM	Acc = 75.3
Saad and Islam, 2019	UMCD	51 ASD, 41 HC	DTI	NA	Graph Theory-based Features	PCA	SVM	Acc = 75 Sen = 81.94 Spe = 70 Pre = 70.42
Liu W. et al., 2020	ABIDE	250 ASD, 218 HC	rs-fMRI	AAL Atlas + CPAC Pipeline	Dimensional Feature Vectors	−	Elastic Net	Acc = 83.33
Zhuang et al., 2018b	Clinical	20 ASD	sMRI	NA	GLM	Different Feature Selection Methods	RF	NA
			rs-fMRI					
Zheng et al., 2019	ABIDE	66 ASD, 66 HC	sMRI	NA	Morphological and MFN Features	RFE	SVM	Acc = 78.63 Sen = 80 Spe = 77.27
ElNakieb et al., 2019	NDAR	122 ASD, 141 HC	DTI	MNI-152 Atlas	Global and Local Feature Extraction	Signal to Noise Ratio (s2n) Filter Based Feature Ranking	SVM	Acc = 71 Sen = 72 Spe = 70
Ge et al., 2018	NDAR	57 ASD, 34 HC	sMRI	NA	Morphometrical Features	−	K-Means Clustering	NA
Stevens et al., 2019	NA	2400 ASD	Different modalities	NA	Latent Clusters	+Bayesian Information Criterion	Linear Regression (LR)	Intensity = 94.29
Wang J. et al., 2020	ABIDE	175 ASD, 234 HC	rs-fMRI	AAL Atlas	Patch-based Functional Correlation Tensor (PBFCT) Features, FC Features	MSLRDA, *T*-test	Multi-View Sparse Representation Classifier (MVSRC)	NA
Dekhil et al., 2019	NDAR	72 ASD, 113 HC	sMRI	Desikan-Killiany (DK) Atlas	Morphological, Volumetric, and Functional Connectivity Features	−	KNN, RF	Acc = 81 Sen = 84 Spe = 79.2
			rs-fMRI					
Abdullah et al., 2019	NA	189 ASD, 515 HC	AQ	NA	Different Features	Chi-Squared Test, LASSO	LR	Acc = 97.54 Sen = 100 Spe = 96.59
Demirhan, 2018	UCI	104 ASD	ASD Scan Data	NA	Different Features	Grid Search Method	RF	Acc = 100 Sen = 100 Spe = 100
Syed et al., 2017	ABIDE	392 ASD, 407 HC	rs-fMRI	DPARSF Pipeline	ICA + Different Features (Reproducible REs, NMI Values, AC Maps)	gRAICAR	K-Means Clustring	Acc = 82.4 Sen = 77 Spe = 87
Liu W. et al., 2020	ABIDE 1	403ASD, 468 HC	rs-fMRI	AAL Atlas + CPAC Pipeline	Dynamic Functional Connectivity (DFC) and Mean Time Series Features	MTFS-EM	SVM	Acc = 76.8 Sen = 72.5 Spe = 79.9
Wang Y. et al., 2020	ABIDE	255 ASD, 276 HC	rs-fMRI	DPARSF Pipeline	Functional Connectivity Features	RFE	SVM	Acc = 90.6 Sen = 90.62 Spe = 90.58
Xiao et al., 2017	Clinical	46 ASD, 39 DD (Developmental Delay)	sMRI	DK Atlas	Neuroanatomical Features (Regional Cortical Thickness, Cortical Volume, Cortical Surface Area)	−	RF	Acc = 80.9 Sen = 81.3 Spe = 81 AUC = 88
Eill et al., 2019	CFMRI	46 ASD, 47 HC	Different Modalities	Johns Hopkins (JH), HO Atlas	Anatomical Variables, Cortical, Mean Diffusivity Values, Connectivity Matrices, and DTI Features	−	Conditional Random Forest (CRF)	Acc = 92.5 Sen = 97.8 Spe = 87.2
Sarovic et al., 2020	Clinical	24 ASD, 21 HC	sMRI	NA	Morphological Features of Subcortical Volumes	−	LR	Acc = 73.2
Zhao F. et al., 2017	ABIDE	54 ASD, 57 HC	sMRI	Different Atlase + DPARSF Pipeline	Regional Morphological Features	HSL-CCA, PCA	Linear SVM	Acc = 81.6 F1-S = 81.4
			t-fMRI					
Dekhil et al., 2018	NDAR	123 ASD, 160 HC	sMRI	All Atlases	PICA (Spatial Components, Correlation Values, Power Spectral Densities)	SAE	SVM	Acc = 92 Sen = 93 Spe = 89
			rs-fMRI					
Yang M. et al., 2019	ABIDE 1	260 ASD, 308 HC	rs-fMRI	AAL Pipeline	−	−	Attention Based Semi-Supervised Dictionary Learning (ASSDL) Model	Acc = 98.2
Jiang et al., 2019	ABIDE 1	250 ASD, 218 HC	rs-fMRI	AAL Atlas + CPAC Pipeline	Multi-Center Domain Adaptation (MCDA) Method	−	KNN	Acc = 73.45 Sen = 69.23 Spe = 79.17
Madine et al., 2020	ABIDE 1	155 ASD, 186 HC	sMRI	DK Atlas	Low-Order Morphological Connectivity Network (LON), Single Cell Interpretation *via* Multi-Kernel Learning (SIMLR), Similarity Matrix	−	Hypergraph Neural Network (HGNN)	Acc = 75.2
Thomas and Chandran, 2018	ABIDE	NA	sMRI	NA	GLCM	−	ANN	NA
			rs-fMRI					
Haweel et al., 2019b	Clinical	30 ASD, 30 HC	t-fMRI	BNT Atlas	GLM Feature Extraction	−	Stacked Non-negativity Constraint Auto-Encoder (SNCAE)	Acc = 75.8 Sen = 74.8 Spe = 76.7
Huang F. et al., 2019	ABIDE 1	109 ASD, 144 HC	rs-fMRI	AAL, Dosenbach 160, CC 200 Atlas + DPARSF Pipeline	Sparse Low-Rank Functional Connectivity Network	Different Feature Selection Methods	SVM	Acc = 81.74 Sen = 71.83 Spe = 89.50
Benabdallah et al., 2018	ABIDE 1	870 Subjects	rs-fMRI	AAL, multi-subject dictionary learning (MSDL) Atlas + CPAC Pipeline	ROIs Extraction, Connectivity Graphs Construction + Minimum Spanning Trees Extraction	MSTs Elimination	SVM	Acc = 74,89 Sen = 24,19 Spe = 93,59
Haweel et al., 2019a	Clinical	30 Subjects	t-fMRI	BNT Atlas	Multi-Level GLM + GLM3 Parameters, Z-Stats Maps for All Brain Areas	RFE	RF	Acc = 78
Alvarez-Jimenez et al., 2020	NDAR	22 ASD, 25 HC	t-fMRI	Proposed Atlas	GLM Analysis	−	Stacked Autoencoder With Non-Negativity Constraint (SNCAE)	Acc = 94.7
			sMRI					
Chaitra et al., 2020	ABIDE 1	34 ASD, 34 HC	sMRI	HO Atlas	Curvelet Transform + Coefficient Distribution Per Curvelet Sub-Band	Generalized Gaussian Distribution (GGD)	SVM	Different Results
	ABIDE II	42 ASD, 41 HC						
Fan et al., 2020	ABIDE 1	432 ASD, 556 HC	rs-fMRI	CC200 Atlas + DPARSF Pipeline	Graph-Theoretic Measures, Traditional FC Data	Recursive-Cluster-Elimination (RCE)	SVM	Acc = 70.1
Mellema et al., 2020	ABIDE 1	145 ASD, 157 HC	rs-fMRI	CC200 Atlas + CPAC Pipeline	Two-Group Cross-Localized Hidden Markov Model	Likelihood Values	SVM	Acc = 74.9
ElNakieb et al., 2018	IMPAC	418 ASD, 497 HC	rs-fMRI	All Atlases	Tangent-Space Embedding Metric	Permutation Feature Importance (PFI)	DenseFFwd	Acc = 75.4–80.4
Ke et al., 2019	Different Datasets	72 ASD, 113 HC	sMRI	DK Atlas	Anatomical and Connectivity Matrix Features	−	KNN, RF, and SVM	Acc = 81 Sen = 78 Spe = 83.5
			rs-fMRI					
Ke et al., 2019	Different Datasets	97 ASD, 56 HC	DTI	JH Atlas	Global Features (FA, MD, AD) + Feature Mapping to Atlas + Local Feature Extraction (PDFs of Features for Each WM Area in the Atlas)	−	KNN, RF, and SVM	Acc = 81 Sen = 78 Spe = 83.5
Mostafa et al., 2019a	NAMIC	2 ASD, 2 HC	sMRI	NA	Adaptive Independent Subspace Analysis (AISA) Method, Texture Analysis + Different Features	t-SNE	KNN	Acc = 94.7 Sen = 92.29 Spe = 94.82 F1-S = 93.56
Bernas et al., 2018	ABIDE 1	403 ASD, 468 HC	rs-fMRI	NA	Eigenvalues and Topology Centralities Features	Backward Sequential Feature Selection Algorithm	LDA	Acc = 77.7
			sMRI					
Dekhil et al., 2020	Clinical	12 ASD, 12 HC	rs-fMRI	NA	Group Independent Component Analysis (gICA) + Wavelet Coherence Maps Extraction	−	SVM	Acc = 86.7 Sen = 91.7 Spe = 83.3
	ABIDE	12 ASD, 18 HC						
Yassin et al., 2020	ABIDE 1	561 ASD, 521 HC	sMRI	DK, AAL Atlas + CCS Pipeline	Anatomical Feature Extraction + Functional Connectivity Analysis	−	KNN	Different Results
			rs-fMRI					
Soussia and Rekik, 2018	Clinical	36 ASD, 106 HC	sMRI	NA	Cortical Thickness, Surface Area, and Subcortical Volume Features	PCA	SVM	Different Results
Xiao et al., 2017	ABIDE 1	155 ASD, 186 HC	sMRI	DK Atlas	Low-Order Morphological Network Construction (LON), High-Order Morphological Network Construction (HON) Features	t-SNE, K-Means Clustering	SVM	Acc = 61.7
Zhao F. et al., 2018	Clinical	46 ASD, 39 DD	sMRI	Talairach, DK Atlas	Regional Cortical Thickness, Cortical Volume, And Cortical Surface Area	−	RF	Acc = 80.9 Sen = 81.3 Spe = 81
Fredo et al., 2018	ABIDE	54 ASD, 46 HC	rs-fMRI	AAL Atlas + DPARSF Pipeline	LON and HONs Features	LASSO	Ensemble Classifier with Multiple Linear SVMs	Acc = 81
Bi et al., 2018	ABIDE	160 ASD, 160 HC	rs-fMRI	HO Atlas	Functional Connectivity Matrix	CRF	SVM	Acc = 65 Sen = 65 Spe = 65
Tejwani et al., 2017	ABIDE	61 ASD, 46 HC	rs-fMRI	AAL Atlas	Graph Theory	−	Random SVM Cluster	Acc = 96.15
Tang et al., 2019	ABIDE	147 ASD, 146 HC	rs-fMRI	CC200 Atlas + DPARSF Pipeline	Two Different Features Sets	−	SVM	Acc = 61.1 Sen = 61.8 Spe = 60
Reiter et al., 2021	ABIDE	42 ASD, 37 HC	rs-fMRI	NA	Functional Connectivity Matrix	−	Different Classifiers	AUC = 97.75
Rane et al., 2017	ABIDE	306 ASD, 350 HC	rs- fMRI	NA	Functional Connectivity Matrix	CRF	RF	Acc = 73.75
Tolan and Isik, 2018	ABIDE 1	539 ASD, 573 HC	rs-fMRI	CPAC Pipeline	Feature Extraction (All Voxels Within Gray Matter Template Mask in MNI152 Space)	−	SVM	Acc = 62
Elnakieb et al., 2020	UMCD	79 Functional and 94 Structural Connectomes	rs-fMRI	NA	Graph Theory + Global, Nodal Measurements, and Gender Information	Relief Algorithm	Ensemble Learning	Acc = 67 pre = 0.67 Recall = 70
			DTI					Acc = 68 Pre = 0.73 Rec = 70
Crimi et al., 2017	NDAR	124 ASD, 139 HC	DTI	JH Atlas	Global and Local Features	Signal to Noise Ratio (S2n) Filter	SVM	Acc = 73 Sen = 70 Spe = 76
Jahedi et al., 2017	ABIDE II	31 ASD, 23 HC	rs-fMRI	AAL Atlas	Connectivity Matrix	−	SVM	Acc = 72.34
			DTI					
			sMRI					
Bhaumik et al., 2018	ABIDE	126 ASD, 126 HC	rs- fMRI	NA	Functional Connectivity Matrix	CRF	SVM	Acc > 90
	Clinical	42 ASD, 30 HC						
Savva et al., 2020	ABIDE	167 ASD, 205 HC	rs-fMRI	CCS Pipeline	Functional Connectivity Matrix	−	SVM	Different Results
Mathur and Lindberg,	ABIDE 1	403 ASD, 465 HC	rs-fMRI	HO Atlas + CPAC Pipeline	sFC, dFC, and Haralick Texture Features	−	SVM	−
Eill et al., 2019	ABIDE	Whole Dataset	rs-fMRI	AAL Atlas + DPARSF Pipeline	Pearson Correlation Coefficient, Graph Measures, and Mean Intensities Features	−	Adaboost	Acc = 66.08
Zhuang et al., 2018a	Clinical	46 ASD, 47 HC	sMRI	JH Atlas	Functional Connectivity Matrix Features	−	CRF	Acc = 92.5 Sen = 97.8 Spe = 87.2
			DWI	HO Atlas				
			rs-fMRI					
Kazeminejad and Sotero, 2019	Clinical	19 ASD	t-fMRI	NA	Elastic Net Regression	−	RF	NA
	ABIDE	64 ASD	rs-fMRI					
[129]	ABIDE 1	816 Subjects	rs-fMRI	AAL Atlas + CPAC Pipeline	Graph Theoretical Metrics	Sequential Forward Floating Algorithm	SVM	Acc = 95 Sen = 97 Spe = 91
Song et al., 2019	ABIDE 1	119 ASD, 116 HC	rs-fMRI	AAL, CC200 Atlas + DPARSF Pipeline	Community Pattern Quality Metrics Features	−	LDA, KNN	Acc = 75 Prec = 76.07 Rec = 71.67
	ABIDE II	97 ASD, 117 HC						
Cordova et al., 2020	Clinical	64 ASD, 66 ADHD, 28 HC	rs-fMRI	NA	43 Executive Functions (EF)	−	Functional Random Forest (FRF)	Different Results
Sadeghi et al., 2017	Clinical	29 ASD, 31 HC	sMRI	Different Atlas	Graph Theory + Different Features	Statistical Analysis	SVM	Acc = 92
		20 ASD, 20 HC	t-fMRI					
Zhang L. et al., 2020	ABIDE 1	21 ASD, 26 HC	rs-fMRI	AAL Atlas + DPARSF Pipeline	Fast Entropy Algorithm + Important Entropy	−	SVM	AUC = 62
Shi et al., 2020	ABIDE 1	59 ASD, 46 HC	rs-fMRI	AAL Atlas + DPARSF Pipeline	Function Connectivity + Minimum Spanning Tree (MST)	−	SVM	Acc = 86.7 Sen = 87.5 Spec = 85.7
Richards et al., 2020	ABIDE 1	437 ASD, 511 HC	sMRI	−	Computing the Brain Asymmetry with The BrainPrint + Asymmetry Values	−	LR Models	NA
Payabvash et al., 2019	Clinical	14 ASD, 33 HC	MRI, DTI	DK Atlas	Different Features	−	Naïve Bayes, RF, SVM, NN	Acc = 75.3 Sen = 51.4 Spec = 97.0
Huang H. et al., 2019	ABIDE	45 ASD, 47 HC	rs-fMRI	AAL Atlas	Modified Weighted Clustering Coefficients	*T*-test and SVM-RFE	Multi-Kernel Fusion SVM	Acc = 79.35 Sen = 82.22 Spec = 76.60
Huang et al., 2020a	ABIDE I	505 ASD, 530 HC	rs-fMRI	CC200 Atlas + CPAC Pipeline	Functional Connectivity	Graph-Based Feature Selection	MMoE Model	Acc = 68.7 Sen = 68.9 Spec = 68.6
Jung et al., 2019	ABIDE,	86 ASD, 83 ADHD, 125 HC	sMRI, rs-fMRI	DK Atlas	Functional Connectivity	Univariate *T*-test and Multivariate SVM-RFE	SVM	Acc = 76.3 Sen = 79.2 Spec = 63.9
DSouza et al., 2019	ABIDE	24 ASD, 35 HC	rs-fMRI	AAL Atlas	Mutual Connectivity Analysis with Local Models (MCA-LM)	Kendall’s τ Coefficient	RF and AdaBoost	Acc = 81
Devika and Oruganti, 2021	ABIDE II	23 ASD, 15 HC	rs-fMRI	AAL Atlas + AFNI Pipeline	Functional Connectivity	ANOVA F-Score	SVM	Acc = 80.76
Ahammed et al., 2021	ABIDE 1	74 ASD, 74 HC	fMRI	DPARSF, CCS Pipeline	Bag-of-Feature (BoF) Extraction	−	SVM	Acc = 81 Sen = 81 Spec = 86
Yap and Chan, 2020	ABIDE	70 ASD, 74 HC	fMRI	NA	Functional Connectivity	Elastic SCAD SVM	SVM	Acc = 90.85 Sen = 90.86 Spec = 90.90
Wang et al., 2019c	ABIDE	250 ASD, 218 HC	rs-fMRI	AAL Atlas + CPAC Pipeline	Functional Connectivity + Low-Rank Representation Decomposition (maLRR)	−	KNN, SVM	Acc = 73.44 Sen = 75.79 Spec = 69.52
Karampasi et al., 2020	ABIDE	399 ASD, 472 HC	rs-fMRI	CC200 Atlas + CPAC Pipeline	Feature Extraction (Static FC, Demographic Information, Haralick Texture Features, Kullback-Leibler Divergence)	Feature Selection Algorithms (RFE-CBR, LLCFS, InfFS, mRMR, Laplacian Score)	SVM, KNN, LDA, Ensemble Trees	Acc = 72.5 Sen = 94 Spec = 64.7
Graña and Silva, 2021	ABIDE	408 ASD, 476 HC	rs-fMRI	CPAC Atlas	5 Methods for Functional Connectivity Matrix Construction	6 Feature Extraction/Selection Approaches	9 Classifiers	−
Yamagata et al., 2019	Clinical	30 Pairs of Biological Siblings	rs-fMRI	Social Brain Connectome Atlas	Functional Connectivity	Sparse LR (SLR)	Bootstrapping Approach	Acc = 75 Sen = 76.67 Spec = 73.33
Conti et al., 2020	Clinical	26 ASD, 24 CAS, 18 HC	sMRI	−	Feature Extraction	Statistical Analysis	SVM	AUC = 73
Deshpande et al., 2013	Clinical	15 ASD, 15 HC	Task-fMRI	−	Functional Connectivity + Effective Connectivity	−	RCE-SVM	Acc = 95.9 Sen = 96.9 Spec = 94.8
Kazeminejad and Sotero, 2020	ABIDE 1	−	rs-fMRI	CC200, AAL Atlas + CPAC Pipeline	Graph Extraction + Feature Extraction	PCA	MLP	Different Results
Song et al., 2019	ABIDE	119 ASD, 116 HC	rs-fMRI	AAL Atlas + DPARSF Pipeline	Resting-State Functional Network Community Pattern Analysis	RFE	LDA	Acc = 74.86 Prec = 76.07 Recall = 71.67
Tang et al., 2019	ABIDE	42 ASD, 37 HC	rs-fMRI	−	Functional Connectivity + Joint Symmetrical Non-Negative Matrix Factorization (JSNMF)	−	SVM	AUC = 97.75
Mhiri and Rekik, 2020	ABIDE	245 ASD, 272 NC	rs-fMRI	DPARSF Pipeline	Different Features	NAG-FS	SVM	Acc = 65.03
Itani and Thanou, 2021	ABIDE 1	201 ASD, 251 HC	rs-fMRI	AAL Atlas + CPAC Pipeline	Graph Construction + Graph Signal Processing (GSP)	Fukunaga-Koontz Transform (FKT)	DT	Acc = 75
Zhan et al., 2021	ABIDE 1	133 ASD, 203 HC	rs-fMRI, sMRI	−	Functional Connectivity	Statistical Analysis	Sparse LR	Acc = 82.14 Sen = 79.70 Spec = 83.74
	ABIDE II	60 ASD, 89 HC						
Wismüller et al., 2020	ABIDE II	24 ASD, 35 HC	rs-fMRI	AAL Atlas	large-scale Extended Granger Causality (lsXGC)	Kendall’s Tau rank correlation coefficient	SVM	Acc = 79
Deshpande et al., 2013	Clinical	15 ASD, 15 HC	fMRI	NA	Functional Connectivity, Effective Connectivity, and Fractional anisotropy (FA) From DTI, Behavioral Scores	Recursive Cluster Elimination	SVM	Acc = 95.9
Jiao et al., 2010	Clinical	22 ASD, 16 HC	MRI	Cortical Atlas	Thickness and Volume-Based Features	Surface-Based Morphometry	Different Classifiers (SVM,FT, LMT)	Acc = 87 Sen = 95 Spe = 75
Ecker et al., 2010b	Clinical	22 ASD, 22 HC	MRI	NA	GLM, Different Features	RFE-SVM	SVM	Spe = 86 Sen = 88
Chen et al., 2013	ABIDE	126 ASD, 126 HC	rs-fMRI	NA	Pearson Correlation Matrix, Connectivity Measures	PSO-SVM	SVM -RFE	Acc = 66 Sen = 60 Spe = 72
Uddin et al., 2011	ABIDE	24 ASD, 24 HC	sMRI	NA	Multivariate Statistical Pattern, Morphological Feature	NA	SVM	Acc = 80
Ingalhalikar et al., 2011	Clinical	45 ASD, 30 HC	DTI	EVE	FA (Fractional Anisotropy), MD Mean diffusivity, Anatomical ROI’s	Signal-To-Noise (s2n) Ratio Coefficient Filter	SVM	Spe = 84 Sen = 74
Varol et al., 2012	Clinical	81 ASD, 50 HC	MRI	NA	Feature Extraction [Voxelwise Tissue Density Maps For GM, WM, and ventricles (VN)]	Welch’s *T*-test	SVM	Acc = 73.28 Sen = 71.6 Spe = 76
Murdaugh et al., 2012	Clinical	13 ASD,15 HC	fMRI	NA	Functional ROIs, Functional Connectivity, Seed-Based Connectivity	*T*-test	Logistic regression	Acc > 96.3
Bloy et al., 2011	Clinical	23ASD,22 HC	MRI	NA	Orientation Invariant Features of Each ROI’s Mean FOD	PCA	SVM	Acc = 77
Giuliano et al., 2013	Clinical	76 ASD,76 HC	sMRI	NA	Sequences Of The Intensity Values Of The GM Segments	SVM-RFE	SVM	Sen = 82 Spe = 80
Deshpande et al., 2013	Clinical	15 ASD, 15 HC	Task-fMRI	NA	Functional Connectivity, Effective Connectivity	NA	RCE-SVM	Acc = 95.9 Sen = 96.9 Spec = 94.8
Ecker et al., 2010a	Clinical	20 ASD, 20 HC	MRI	NA	Morphological Parameters Including Volumetric and Geometric Features	NA	SVM	Sen = 90 Spe = 80
Li et al., 2012	Clinical	10 ASD,10 HC	DTI	JHU-DTI-MNI	Brain Connectivity Network	Network Regularized SVM-RFE	SVM	Acc = 100
Bryant et al., 2012	Clinical	31 Klinefelter syndrome, 8 XYY Syndrome 75 HC	sMRI	NA	Statistical Parametric Mapping [Gray Matter Volume (TGMV) A Volume (TWMV) Measures]	RFE	SVM	NA
Vigneshwaran et al., 2013	Clinical, ABIDE	79 ASD,105 HC	MRI	NA	Voxel Locations of VBM Detected Brain Region	*T*-test	PBL-McRBFN	Acc (Mean) = 70 Sen (Mean) = 53 Spe (Mean) = 72
Sato et al., 2013	Clinical	82 ASD, 84 HC	sMRI	NA	Inter-Regional Thickness Correlation (IRTC) Using Pearson Correlation Between the Cortical Thicknesses of Each Region.	NA	Support Vector Reression	NA
An et al., 2010	Clinical	DTI Data: 5 b0 iImages, followed by 30 Diffusion Weighted Images, Child Control dataset	fMRI	Brodmann	Fiber Connectivity Feature, ROIs Extraction, Functional Connectivity Information	NA	mv-EM	Max Percent Error: mv-EM: 8.55
			DTI					
Sadato and Tanabe, 2012	Clinical	21 ASD,21HC	fMRI	NA	Neural Substrates And Inter-Individual Functional Connectivity	*T*-test	NA	Acc = 74.2∓1.9
Filipovych et al., 2012	BLSA	17 MCI (mild cognitive impairment)	MRI	NA	Tissue Density Maps, Top-Ranked Features Wavelet Decomposition Level	Wavelet-Based Data Compression	JointMMCC	Different Results
Calderoni et al., 2012	Clinical	38 ASD, 38 HC	sMRI	NA	Volumetric Variables (GM, WM, CSF, TIV),	SVM-RFE, *T*-test	SVM	AUC = 80
Jiao et al., 2011	Clinical	13 ASD	MRI	NA	Regional Cortical Thicknesses And Volumes	NA	Three Decision-Tree-Based Models, SVM, logistic Model Tree	Acc > 80 Spe > 34 Sen > 92
Nielsen et al., 2013	ABIDE	447 ASD, 517 HC	rs-fMRI	NA	Functional Connectivity From a lattice of ROIs Covering The Gray Matter	NA	leave-one-out	Acc = 60 Spe = 58 Sen = 62
Jiao and Lu, 2011	Clinical	22 ASD, 16 HC	MRI	NA	Using Surface-based morphometry For Cortical Features (Average thickness, Mean Curvature, Gaussian curvature, Folding index, Curvature index)	NA	SVM,FT,LMT	Acc (SVM) = 74 Acc (FT) = 76 Acc (LMT) = 76
Retico et al., 2016a	Clinical	76 ASD, 76 HC	sMRI	NA	GM Volumes	RFE	SVM	AUC = 82
Retico et al., 2016b	Clinical	41 ASD, 40 HC	sMRI	NA	Regional Features	−	SVM	AUC = 81
Subbaraju et al., 2017	ABIDE	505 ASD, 530 Neurotypical Subjects	rs-fMRI	NA	Spatial Feature-based Detection Method (SFM) (Mean Connectivity Matrices, Discriminative Log-variance Features)	Feature Selection Based on top m Signals	SVM	Acc = 95
Gori et al., 2016	Clinical	41 ASD, 40 HC	sMRI	NA	ROI Features	−	SVM	AUC = 74
Lu et al., 2015	Clinical	35 ASD, 51 TD, 39 No Known Neuropsychiatric Disorders	fMRI	NA	Individual Difference Measures in BOLD Signals	−	LR	Sen = 63.64 Spe = 73.68
Chen et al., 2016	ABIDE	112 ASD, 128 HC	rs-fMRI	NA	Functional Connectivity Values	F-score Method	SVM	Acc = 79.17
Wee et al., 2014	NDAR	58 ASD, 59 HC	sMRI	NA	Regional and Interregional Morphological Features	*T*-test	SVM	Acc = 96.27 AUC = 99.52
						mRMR		
Zhou et al., 2014	ABIDE	127 ASD, 153 TD	sMRI	NA	Quantitative Imaging Features (Regional Gray Matter and Cortical Thickness Volumes)	mRMR	SVM	Acc = 70

### Preprocessing techniques for functional and structural modalities

**FIGURE 3:**
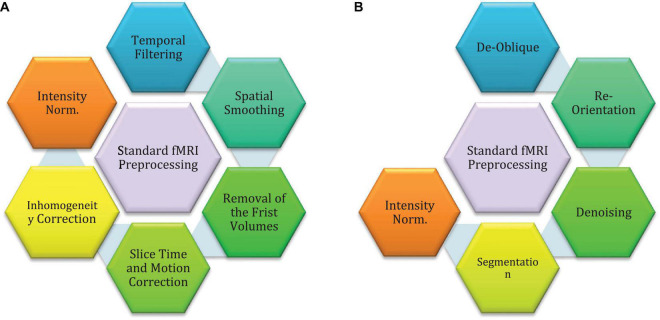
Standard preprocessing methods for MRI neuroimaging modalities: **(A)** preprocessing for fMRI data, **(B)** preprocessing for sMRI data.

Preprocessing techniques are needed to help CADS to achieve promising results. The sMRI and fMRI neuroimaging modalities have implemented fixed preprocessing steps using different software packages. The most common software packages are brain extraction tools (BET) (Soltaninejad et al., 2014), FMRIB software libraries (FSL), statistical parametric mapping (SPM), and FreeSurfer (Khodatars et al., 2021). The following is the standard preprocessing steps for fMRI and sMRI neuroimaging modalities. Some of them are common for both fMRI and sMRI modalities, so we will introduce them in the fMRI-related section. Figure 3 shows the standard fMRI and sMRI techniques. Also, the pipeline preprocessing techniques for ABIDE datasets will be introduced in another section.

The standard Preprocessing is a necessary step in fMRI, and if preprocessing is not carried out properly, it will lead to reduced performance of automated diagnosis of ASD. This step aims to extract regions suspected of having ASD and examine the function of brain neurons in those regions. The preprocessing steps of fMRI include delineation of the brain region, removal of the first few volumes, slice timing correction, inhomogeneity correction, motion correction, intensity normalization, temporal filtering, spatial smoothing, and ultimately registration standard atlas (Khodatars et al., 2021). As mentioned earlier, this step is usually carried out by a toolbox, including BET (Khodatars et al., 2021), FSL (Khodatars et al., 2021), SPM (Anand and Sahambi, 2010; Khodatars et al., 2021), FreeSurfer (Lee and Xue, 2017; Khodatars et al., 2021), etc. In reference (Khodatars et al., 2021), the details for standard preprocessing steps of fMRI modalities are elaborately explained.

The preprocessing of sMRI data extracts helps physicians examine regions with suspected ASD more accurately. Besides, low-level sMRI preprocessing methods help AI-based CADS to process important information. This process increases the accuracy and efficiency of diagnosis of ASD CADS. The most important standard sMRI covers intensity standardization, de-oblique, re-orientation, Denoising, and segmentation (Khodatars et al., 2021). In reference (Khodatars et al., 2021), each step of standard preprocessing for sMRI modalities is explained.

#### Pipeline methods

The pipelines are a preprocessed version of ABIDE data using standard preprocessing procedures, which researchers can use to avoid the problems of variations in the output between different studies as a result of preprocessing. The most popular ABIDE pipelines include neuroimaging analysis kit (NIAK), data processing assistant for rs- fMRI (DPARSF), the configurable pipeline for the analysis of connectomes (CPAC), and connectome computation system (CCS) (Khodatars et al., 2021).

### Feature extraction

Representing data that allows ML algorithms to reason about them is the key to any related research. If this is not done, high performance cannot be achieved. Most commonly used feature extraction schemes for fMRI and sMRI are statistical, texture, morphological, non-linear, graph, functional connectivity, and structural connectivity schemes.

#### Statistical features

Autism spectrum disorder is typically detected with MRI modalities using statistical features, the most straightforward group of features. Despite their simplicity, these features are usually informative and can also serve as a benchmark for evaluating other methods of feature extraction as well. Additionally, the process of extracting these features is not time-consuming in comparison to other methods. However, these methods do not reveal non-linear or subtle patterns in data. Using statistical features for ASD diagnosis, Dekhil et al. (2019) extracted various statistical features from MRI data and then applied KNN and SVM algorithms in the classification step. The authors reported 81% accuracy.

#### Texture features

As a group of features, spatial patterns form an indispensable group, possibly the most important group, since the cognitive system of the human is mostly dependent on them. Gray-level co-occurrence matrix (GLCM) (Jafarpour et al., 2012) feature extraction is one of the most widely used methods in various research studies (Thomas and Chandran, 2018) among various textures-based features. Haweel et al. (2020) presented an ASD diagnostic method based on MRI data. Texture features and the RFE technique were used in the feature extraction and feature selection steps. Then, the authors used the RF technique for classifying features and reached an accuracy of 72%. In another study, scholars used various methods, such as Haralick, in the feature extraction step from sMRI data. Then, the authors tested different feature selection methods and reached an accuracy of 72.5%.

#### Morphological features

Morphological operation is an important feature extraction technique frequently used in image processing (Usha and Perumal, 2019). In these methods, features are extracted from the appearance and shape of the image. Morphological operation is often used to process binary images, but they can also be used for gray and color-level images (Gupta et al., 2019). Morphological features are also commonly used for diagnosing brain diseases from sMRI modalities. Zheng et al. (2019) proposed the idea of ASD diagnosis using morphological features. After feature extraction, RFE and SVM were tested for feature selection and classification, respectively. An accuracy of 78.63% was obtained.

#### Non-linear features

A non-linear characteristic of biological data is emphasized when considering non-linear features. The performance of CADS for ASD is significantly enhanced through the use of these features (Anand and Sahambi, 2010). In reference (Mellema et al., 2020), non-linear-based features of likelihood are used to detect autism using MRI neuroimaging methods. Entropies are one of the most important non-linear methods that are widely used to extract features from signals and brain images (Georgiadis et al., 2008). Functional imaging modalities are non-linear and chaotic, which has led researchers to use entropy-based non-linear features to diagnose brain disorders (Saritha et al., 2013; Wang et al., 2022). Zhang L. et al. (2020) introduced a novel ASD diagnostic method using fMRI data and a new entropy method. This study initially used fast entropy for feature extraction from preprocessed fMRI data. Then, they used the SVM algorithm for feature classification and obtained satisfactory results.

#### Graph features

This group of features is highly relevant to the analysis of MRI data. Graph-based features are derived first by shaping the data into a graph, and then, from the constructed graph, local and global features are extracted (Lee and Xue, 2017). Researchers have explored graph features to diagnose ASD using fMRI data in many studies. Bi et al. (2019) employed rs-fMRI from the ABIDE database for ASD diagnosis using graph and genetic-evolutionary random SVM cluster (GERSVMC) for feature extraction and classification steps, respectively, and obtained an accuracy of 62%. Saad and Islam (2019) presented an ASD diagnostic method based on graph features in another study. After feature extraction *via* the graph method, PCA and SVM techniques were used for feature reduction and classification, which resulted in an accuracy of 75% for ASD diagnosis.

#### Connectivity matrix

In order to process sMRI and fMRI neuroimaging images, feature extraction methods based on connectivity matrix methods are typically employed (Zeng et al., 2018; Yeh et al., 2021). Such features provide information about the brain’s structure and function. The functional connectivity matrix (FCM) (Zhou and Wang, 2007; Yan and Zhang, 2015) and structural connectivity matrix (SCM) (Yang et al., 2016; Ma et al., 2019) are the measures employed for fMRI and sMRI modalities, respectively. Connectivity features are mostly used in diagnosing brain disorders. Table 2 and Supplementary Table 1 outline studies on ASD diagnosis from MRI modalities using various AI techniques. Table 2 shows that connectivity methods are most frequently used for feature extraction from MRI modalities. Liu W. et al. (2020) used dynamic functional connectivity (DFC) in the feature extraction step of rs-fMRI data. The feature selection step was also conducted by the MTFS-EM method. Finally, they used the SVM method for classification and obtained an accuracy of 76.84%. In another study, Mathur and Lindberg, utilized DFC and static functional connectivity (SFC) in the feature extraction step. Then, the SVM was tested for connectivity-based classification of features. Authors could finally obtain satisfactory results for ASD diagnosis using connectivity features.

### Feature reduction/selection methods

It has been shown that increasing the number of extracted features can help algorithms to represent data in a more meaningful and robust way; however, the curse of dimensionality (Fodor, 2002) may cause it to backfire and reduce performance. Several methods for reducing dimensionality and selecting features have been proposed to prevent this from occurring. In addition, these methods are widely used to increase the performance of CADS for detecting autism spectrum disorders. Several methods have previously been used in research papers, including principal component analysis (PCA) (Wold et al., 1987), recursive feature elimination (RFE) (Yan and Zhang, 2015), *T*-test (Zhou and Wang, 2007), autoencoder (AE) (Yang et al., 2016), conditional random forest (CRF) (Ma et al., 2019), Chi-squared (Ye and Yang, 2010), and least absolute shrinkage and selection operator (LASSO) (Muthukrishnan and Rohini, 2016). The following is a brief description of these methods.

#### Principal component analysis

Principal component analysis is arguably the most common dimensionality reduction method (Wold et al., 1987). It works by finding and representing data by the principal components, i.e., the vectors that preserve the most data variance. One of the benefits of PCA is its ability to find a minimal number of features required to preserve a specified variance ratio (Wold et al., 1987). Principal component analysis (PCA) is one of the most popular feature reduction techniques in medical applications and has also been used in ASD diagnosis research (Zhao F. et al., 2017; Soussia and Rekik, 2018; Saad and Islam, 2019; Kazeminejad and Sotero, 2020).

#### Recursive feature elimination

Recursive feature elimination is more of a wrapper-type algorithm, meaning that it is applied to a classification algorithm to find the best subset of features. As the name explains, this algorithm works by eliminating features one by one to reach the optimal number. First, a classification algorithm is trained on the dataset, ranking feature importance’s. The least important feature is then eliminated and repeated until the number of remaining features matches the desired number (Yan and Zhang, 2015). Haweel et al. (2020) proposed a novel ASD diagnostic method using the GLM feature extraction technique. After feature extraction from MRI data, the RFE technique was used for feature reduction. The RF method was also tested in the classification step with an accuracy of 72%.

#### *T*-test

To find the best set of features, *T*-test calculates a score for each feature, then ranks them based on that score and picks the top features as selected ones. The score shows whether the values of a feature for a class are significantly different from those for another class by calculating the mean and standard division (STD) of each feature in each class (Zhou and Wang, 2007). A new ASD diagnostic method from MRI data was introduced by Sartipi et al. (2018). First, the graph technique was used for feature extraction from sMRI modalities. Then, they applied the *T*-test and SVM algorithms for the feature selection and classification steps and acquired an accuracy of 75%.

#### Chi-squared

Chi-Square is suitable when the features are categorical, and the target variable is also categorical, such as classification. Chi-Squared measures the degree of association between two variables; thus, features that connect with the targets can be picked (Ye and Yang, 2010). When the features are numerical, we can use a *T*-test, or Chi-Square can be used for the numerical variable by discretizing them (Ye and Yang, 2010). In reference (Dekhil et al., 2019). The authors proposed a new ASD diagnostic method using various ML techniques from MRI data. Various methods were used for feature extraction. Then, the Chi-squared method was tested for the feature selection step. Next, the LR classification algorithm was applied, which resulted in a promising performance.

#### Least absolute shrinkage and selection operator

Least absolute shrinkage and selection operator is mainly a regression method; however, this algorithm can also be used for feature selection (Muthukrishnan and Rohini, 2016). Notably, linear regression with L1 regularization is called Lasso. After training, the lasso assigns a weight to each feature for the regression (Muthukrishnan and Rohini, 2016). Using these weights, there are two methods to pick the best features, first, pick the K highest valued weights; second, pick all the weights which have a value higher than a specified threshold (Muthukrishnan and Rohini, 2016). Fredo et al. (2018) proposed a new ASD diagnostic method based on Hons and Lon features. Their paper used LASSO and SVM methods for feature selection and classification. They reported an accuracy of 81%.

### Classification methods

This section discusses the various classification algorithms used in CADS for ASD. As mentioned earlier, classification is the last step in a CADS based on ML methods. Support vector machine (SVM) (Noble, 2006; Suthaharan, 2016), linear discriminant analysis (LDA) (Zhang et al., 2007), k-nearest neighbor (KNN) (Liao and Vemuri, 2002), and random forest (RF) (Oshiro et al., 2012) are arguably among the most popular methods used in CADS created for ASD. Table 2 and Supplementary Table 1 show the classification algorithms used for ASD detection. A summary of classification algorithms used for automated detection of ASD are presented below.

#### Support vector machine

Support vector machines are among the oldest classification and has been widely used in many applications (Noble, 2006; Suthaharan, 2016). SVM tries to find the best hyperplane to separate data points; however, it only needs the dot product between every two data points (Noble, 2006; Suthaharan, 2016). Consequently, to transform data into another space, only a function that gives the dot product of two points in that space would suffice; this is also named kernel trick and is used widely in other fields. Using an appropriate kernel, SVM can usually yield high classification performances (Noble, 2006; Suthaharan, 2016).

#### Random forest

Random forests are an ensemble learning-based method proposed to make the decision trees robust to outliers (Oshiro et al., 2012). The basic idea is to train many trees and determine the final output based on voting among their outputs. To make the final results robust, each tree is trained only on a fraction of the data, and also each tree sees a fraction of all features. The picked ratio for both of these is the square root of the available number.

#### Linear discriminant analysis

Used as a tool for dimension reduction, classification, and data visualization (Zhang et al., 2007). It is simple and robust and yields interpretable classification results (Zhang et al., 2007). It works by dividing the data space into *K* disjoint regions that represent all the classes; then, in the testing phase, the label is determined by finding the region in which the data belongs. LDA can be used as the first benchmarking baseline before other, more complicated ones are employed for real-world classification problems (Zhang et al., 2007).

#### K-nearest neighbor

This classifier is among the simplest yet efficient algorithms; its main idea is to assign the label of each data point based on the label of those closest (Liao and Vemuri, 2002). Consequently, there is no training phase; however, for each test subject, the distance to all training points must be calculated, which scales with the size of the dataset; thus, this method is not applicable to enormous datasets. After finding the closest points, the final label is determined using a voting scheme (Liao and Vemuri, 2002).

## Challenges in detecting autism spectrum disorder with magnetic resonance imaging neuroimaging modalities and artificial intelligence techniques

This section introduces the challenges facing ASD detection from MRI neuroimaging modalities and AI techniques. The challenges mentioned in this section cover dataset limitations, lack of access to multimodal datasets, AI techniques, and suitable hardware resources. They are briefly described below.

### Unavailable magnetic resonance imaging neuroimaging datasets with different autism spectrum disorder patient

All datasets available involve two classes of ASD and control fMRI or sMRI modalities (Heinsfeld et al., 2018; El-Gazzar et al., 2019; Felouat and Oukid-Khouas, 2020). However, there are different types of ASD, and this poses a serious obstacle for researchers in AI wishing to develop systems that can detect different types of disorders. Datasets with different types of ASD can help pave the way for accurate diagnosis of various types of ASD.

### Unavailable multi-modalities datasets for autism spectrum disorder diagnosis

In medical research, specialists have shown that neuroimaging multimodalities can effectively improve diagnosis of brain disorders. Neuroimaging modality fusion is one of the newest methods for diagnosing brain disorders such as ASD (Jones et al., 2011), SZ (Bora et al., 2011), and ADHD (Sibley et al., 2022). Physicians usually use MRI data with other neuroimaging modalities to diagnose brain disorders. To diagnose neurological and mental disorders, fMRI-MEG (Kober et al., 1993), MRI-PET (Loeffelbein et al., 2012), and EEG-fMRI (Valdes-Sosa et al., 2009) are the most important multimodalities. Unfortunately, the neuroimaging multimodalities datasets are not available for studies on ASD diagnosis. Such datasets might lead to practical and interesting studies in ASD diagnosis.

### Challenges in artificial intelligence algorithms in diagnosing autism spectrum disorder

Computer-aided design systems based on ML algorithms are highly time-consuming and complex to design. However, if the appropriate algorithms are selected, it can accurately diagnose ASD (Iglesias et al., 2017; Khosla et al., 2019; Hiremath et al., 2020; Leming et al., 2020, 2021). DL methods automatically perform the steps from feature extraction to classification. By using intelligent feature extraction, DL eliminates the need for supervision on features, which may reduce the performance of a CADS based on DL compared to ML. Therefore, when ML methods are combined with DL, promising results can be obtained in CADS for the diagnosis of ASD.

### Challenges in hardware’s

The lack of access to appropriate hardware resources is another problem encountered by researchers in the field of automated ASD detection. ASD detection datasets that are available publicly, such as ABIDE, have a lot of data; this poses many challenges for storing and processing these datasets on ordinary computers. In contrast, research in CAD implementation on cloud servers has not been seriously conducted to eliminate hardware resource problems. As a result, cloud servers are not yet extensively used for data storage and processing. Recently, some DL models called deep compact CNN models have been introduced to be implemented on hardware systems with limited resources (Zhang Z. et al., 2020). Deep compact-size CNN models require fewer hardware sources than other CNN methods (Tian et al., 2018; Wong et al., 2019). Some deep compact-size CNN methods include FBNetV3 (Srinivas et al., 2019), MobileNet (Michele et al., 2019), and TinyNet (Wu et al., 2018).

## Discussion

This paper presents and compares the research about automated ASD detection with MRI neuroimaging modalities and AI methods. First, this section comprehensively compares the conducted studies on ASD detection using ML and DL techniques. In subsection one, the number of studies conducted annually in ASD detection from MRI neuroimaging modalities using different ML and DL techniques is presented. In subsection two, the MRI datasets employed in studies on the automated diagnosis of ASD using ML and DL techniques are compared. In subsection three, the number of MRI studies conducted annually on ASD detection from MRI neuroimaging modalities is discussed. The employed atlas in ML and DL studies for ASD detection is introduced in subsection four. Finally, section five discusses MRI pipeline techniques in the diagnosis of ASD research using ML and DL methods. Ultimately, different classification algorithms for ML and DL-based diagnosis of ASD are compared.

### Comparison between the numbers of papers published each year for machine learning and deep learning research

**FIGURE 4:**
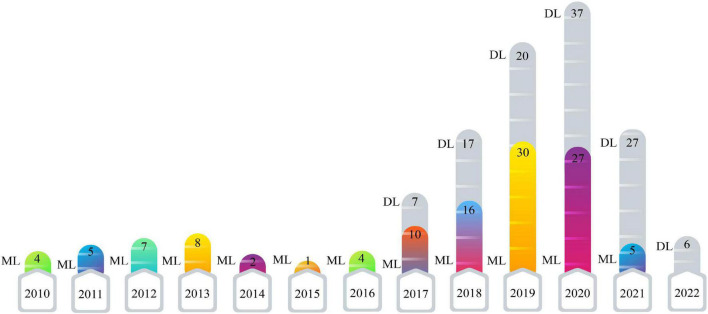
Shows the number of papers published in ASD detection using ML and DL methods.

This section presents the number of published papers annually on ASD detection using AI techniques. Studies on the ASD detection from MRI modalities and ML and DL techniques began in 2017. Table 2 represents the papers on ASD detection in MRI neuroimaging modalities using ML methods. In addition, articles in ASD detection in MRI neuroimaging modalities using DL techniques are introduced in Supplementary Appendix A. Figure 4 illustrates the number of papers published annually on ML and DL techniques for ASD detection.

As demonstrated in recent years, researchers’ interest in using DL architectures has significantly grown compared to ML techniques. According to Figure 4, DL models are used more in studies on the automated diagnosis of ASD with MRI modalities than ML models. Therefore, implementing CADS based on DL techniques is promising for developing applied software for ASD detection with MRI neuroimaging modalities in the future. For automated diagnosis of ASD with MRI modalities, various datasets are proposed in ABIDE. Besides, various toolboxes are available for the implementation of different DL models. These reasons are the foundation for many studies on the automated diagnosis of ASD using DL models.

### Comparison between the numbers of datasets used in the machine learning and deep learning research

**FIGURE 5:**
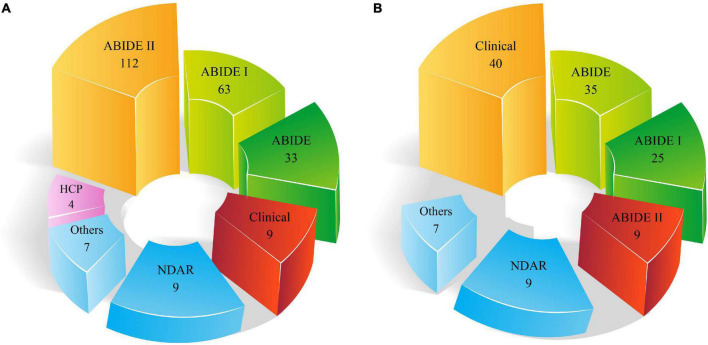
Number of datasets used for automated ASD detection. **(A)** DL and **(B)** ML methods.

As stated in the neuroimaging modalities section, limited datasets are accessible. ABIDE is the most important dataset available in this field, which includes two datasets, ABIDE I and ABIDE II. Figure 5 demonstrates the types of datasets employed in the automated ASD diagnostic research using DL and ML techniques.

It can be noted from Figures 5A,B that a greater number of ABIDE datasets are employed in studies on the automated diagnosis of ASD. The major reason for the wide use of this dataset in various studies on the automated diagnosis of ASD is the availability of many subjects and different MRI modalities.

### Comparison between the numbers of neuroimaging modalities used in the machine learning and deep learning research

**FIGURE 6:**
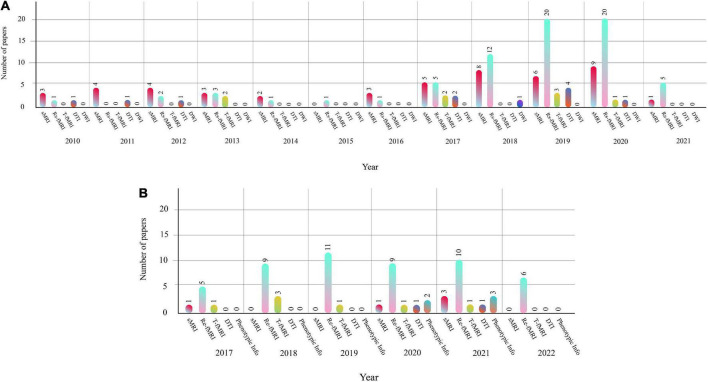
**(A)** Shows the number of MRI neuroimaging modalities used in the CADS based on ML methods. **(B)** Shows the number of MRI neuroimaging modalities used in the CADS based on DL methods.

The different structural and functional MRI neuroimaging modalities and ML and DL methods play an essential role in automated ASD detection. Table 2, reports studies on automated ASD detection using ML techniques and different MRI neuroimaging modalities have been presented. Moreover, Supplementary Table 1 discusses ASD detection using DL techniques. Figures 6A,B describes the annual research carried out to detect automated ASD using sMRI and fMRI neuroimaging modalities.

As shown in Figures 6A,B, the rs-fMRI modalities are most used in studies on ASD detection using ML and DL methods. As mentioned earlier, ASD is a neurological disorder that negatively affects brain function. Accordingly, researchers have used rs-fMRI modalities most widely in studies on ASD detection using AI methods.

### Comparison between the numbers of atlases used in the machine learning and deep learning research

**FIGURE 7:**
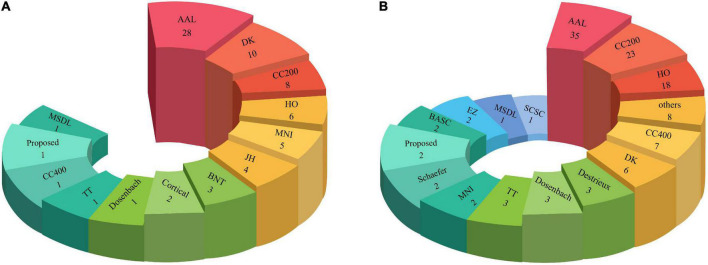
Number of Atlas used for ASD detection. **(A)** ML and **(B)** DL methods.

In another part of Table 2 and Supplementary Table 1, the types of Atlases for MRI neuroimaging modalities have been provided. Atlases are considered an important preprocessing step discussed in part of this section. The number of atlases employed in ML and DL research are described in Figure 7.

As shown in Figures 7A,B, the AAL atlas is most used in studies for ASD detection in MRI neuroimaging modalities using AI methods.

### Comparison between the numbers of pipelines used in the machine learning and deep learning research

**FIGURE 8:**
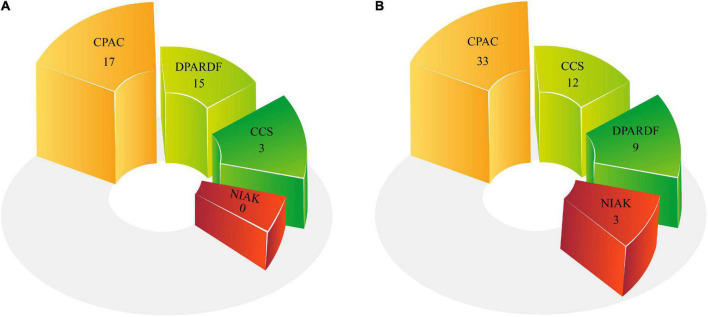
Number of pipelines used for ASD Detection: **(A)** ML and **(B)** DL methods.

Pipelines play a significant role in preprocessing of MRI modalities. The pipelines employed in ASD data preprocessing are presented in Table 2 and Supplementary Table 1. The number of pipelines utilized in DL and ML research is shown in Figure 8. The results of the studies reveal that the CPAC pipeline is the most widely used.

### Comparison between the numbers of classification methods in the machine learning and deep learning research

**FIGURE 9:**
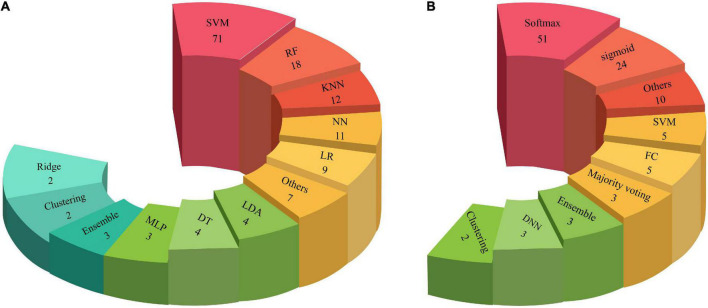
Number of classifiers used in CADS for ASD detection: **(A)** ML and **(B)** DL methods.

Classification is the last step of CADS with ML or DL methods. So far, various classification methods have been proposed in ML and DL, presented in Table 2 and Supplementary Table 1. The types of classification algorithms applied in CADS using DL and ML are depicted in Figure 9. As shown in this Figures 9A,B, it may be noted that the Softmax method is most used in DL architectures. In addition, SVM is the most widely applied in ML methods compared to other classification methods.

## Future works

Lack of access to huge public datasets with various ASD disorders researchers is a big challenge. As mentioned in the introduction, autism has different types (Sparks et al., 2002), and the availability of datasets containing different types of ASD is of paramount importance for researchers. Hence, presenting MRI datasets of different types of autism disorder need to be addressed in future works. These datasets help researchers conduct more studies and compare their studies with other researchers on the automated diagnosis of ASD. As mentioned in previous sections, ABIDE is a free dataset available for researchers and consists of different cases and MRI modalities of ASD patients. But it does not have many cases of DTI modalities for the diagnosis of ASD. DTI modality is one of the popular methods in ASD detection. Publicly providing more datasets of this type of modality could increase research in the ASD diagnosis field using the DTI modality.

Another future work is to provide multimodal datasets, such as fMRI-EEG, for the diagnosis of ASD. In clinical studies (Cociu et al., 2017), it has been indicated that using multimodal neuroimaging, such as fMRI-EEG, plays a pivotal role in diagnosing ASD. In addition, providing datasets with combined modalities paves the way for new studies on the diagnosis of ASD using different AI methods.

Automated diagnosis of ASD with MRI using ML techniques can be the other future work. Various methods have been proposed for feature extraction from MRI data for the diagnosis of ASD, which are summarized in Table 2. According to Table 2, fuzzy-based feature extraction techniques have not been used in the diagnosis of ASD, and they can be introduced in future work. Fuzzy techniques are important in medical applications and allow researchers to develop software close to human logic (Chanussot et al., 1999; Davidson et al., 2001; Javed et al., 2013; Jiang et al., 2017; Meena and Agilandeeswari, 2020; Ullah et al., 2020). Hence, providing graph models based on fuzzy theory can be addressed in the future, leading to the accurate diagnosis of ASD with MRI modalities. Connectivity techniques are an essential feature extraction method for structural and functional neuroimaging modalities (Bhattacharya et al., 2006; Rowe et al., 2010; Smith et al., 2012; Gilson et al., 2018; Park et al., 2018; Zarghami and Friston, 2020). Proposing new feature extraction methods based on connectivity for structural and functional neuroimaging modalities is another field for future work. Table 2 also indicates classification algorithms. In this section, fuzzy type 1 and 2 techniques can be used for data classification as future work on the diagnosis of ASD (Melin and Castillo, 2013, 2014; de Aguiar et al., 2017). Furthermore, in the future, graph theory-based classification methods can also be used to increase the performance of the CADS for automated diagnosis of ASD (Cai et al., 2018; Wu et al., 2020).

The reliability of AI models for medical diagnosis (Balagurunathan et al., 2021; Durán and Jongsma, 2021) poses another challenge for researchers, which needs to be solved before these models are usable in real-life. There is more than one direction that contributes toward this end, such as designing test and validation protocols to ensure the validity of reported results, necessitating papers to include enough information to make results reproducible (such as protocols used in top-tier conferences such as NeurIPS) and also working on explainability and interpretability of models in addition to their performances (Afnan et al., 2021).

In Supplementary Appendix (A), different studies on the automated diagnosis of ASD using MRI modalities and DL techniques is presented. It may be noted that conducted studies have used standard DL methods to diagnose ASD. In future works, graph theory (Zhang Z. et al., 2020; Ma et al., 2021), representation learning (Hamilton et al., 2017; Zhang et al., 2018), zero-shot learning (Wang et al., 2019d), Q-learning (Jang et al., 2019), attention learning (Li et al., 2018c), and advanced models of adversarial networks (Liu and Tuzel, 2016; Creswell et al., 2018) can be used for the automated diagnosis of ASD with MRI modalities.

Feature fusion technique is a new field in diagnosing different diseases, and many studies are being conducted in this field (Antropova et al., 2017; Fan et al., 2018; Hermessi et al., 2019; Liu et al., 2021; Wang et al., 2021b; Amemiya et al., 2022). The DL features can be extracted from MRI images for automated ASD detection. Ultimately, ML and DL features can be used to obtain high performance in the automated diagnosis of ASD.

## Conclusion

Autism spectrum disorder is a neurological disorder with unknown symptoms that begins in childhood and cause problems in communication, social relationships, perception processing, and repetitive behaviors. In few studies, physicians have stated that ASD usually occurs due to genetic mutations or the inability of the fetus’s brain cells to obey regular growth patterns during the first steps (Sparks et al., 2002; Brieber et al., 2007; Sato et al., 2012; Ecker et al., 2015; Hernandez et al., 2015).

Physicians use different ASD detection methods, among which different neuroimaging modalities are of paramount importance (Parisot et al., 2018; Mellema et al., 2019; Ronicko et al., 2020). Among different neuroimaging modalities, MRI-based functional and structural modalities are mostly used to diagnose ASD. sMRI and fMRI provide physicians with important information on the structure and function of the brain, respectively (Sserwadda and Rekik, 2021; Tummala, 2021). However, accurate diagnosis of ASD from sMRI and fMRI is sometimes time-consuming and challenging. Moreover, factors such as tiredness or different noises in MRI modalities may lead to clinicians’ wrong diagnosis of ASD.

For this purpose, many studies are being conducted on the automated diagnosis of ASD using AI techniques, aiming to increase the performance of automated diagnosis of ASD. In general, studies on the automated diagnosis of ASD from MRI modalities using AI cover ML and DL methods. In few papers, researchers have conducted a review study in ASD detection based on DL (Khodatars et al., 2021) and ML (Brihadiswaran et al., 2019; de Belen et al., 2020; Hosseinzadeh et al., 2021; Kollias et al., 2021; Song et al., 2021; Tawhid et al., 2021) methods with different neuroimaging modalities.

This work is a comprehensive review of studies conducted on ASD detection using AI methods in different MRI neuroimaging modalities. First, AI-based CADS for ASD detection from different MRI neuroimaging modalities was introduced. Then, the steps of the CADS based on ML algorithms for automated ASD detection in MRI neuroimaging modalities were studied. Also, in this section, papers on the automated ASD detection in MRI neuroimaging modalities using ML methods are summarized in Table 2. Previously, some authors of this study previously published a review paper about automatic ASD detection in different neuroimaging modalities using DL techniques (Khodatars et al., 2021), which is summarized in Supplementary Table 1.

The most critical challenges in ASD detection in MRI neuroimaging modalities and AI methods were presented in another section. Also, this section studied the most important challenges in the automated diagnosis of ASD using MRI modalities and AI techniques. The most important challenges in the diagnosis of ASD are the lack of access to public datasets with different MRI modalities, multimodal datasets, such as fMRI-EEG, AI algorithms, and hardware resources.

In the discussion section, first, the number of published annual papers on ASD detection using ML methods and DL techniques were discussed. Then, the number of datasets used in ML and DL studies was presented. In addition, the number of different MRI neuroimaging modalities with ML and DL methods used in annual studies in ML and DL was also indicated. Also, a comparison was made between different atlases used in MRI neuroimaging preprocessing for ASD detection. In another subsection, the number of pipelines in the preprocessing step of the MRI neuroimaging modalities for CADS based on various AI methods is also examined and compared. Finally, the number of classifier algorithms used in ML and DL studies for ASD detection was discussed.

In section 7, the future works for ASD detection in MRI neuroimaging modalities and AI methods were addressed. In this section, future works on MRI datasets for the diagnosis of ASD were first discussed. Then, future works on the diagnosis of ASD using AI techniques were addressed. Besides, future works on the automated diagnosis of ASD with MRI modalities were introduced. The final section also recommended the idea of using feature fusion for the diagnosis of ASD with MRI modalities in future works. Studies on ASD detection using AI techniques indicate that researchers will use the proposed methods in the future. The proposed methods are promising in developing real software for ASD detection using MRI modalities and help clinicians quickly diagnose ASD in the early stage.

Also, research on DL-based methods for the diagnosis of ASD has experienced significant attention in recent years. In standard mode, sMRI and fMRI data are recorded in 3D and 4D. However, in most papers, researchers have utilized 2D DL models to diagnose ASD using MRI neuroimaging modalities. Due to the high computational cost of 3D DL models for diagnosing ASD, there has been less research in this field. Providing 3D DL models based on quantization techniques reduces hardware resources and increases speed. Thus, DL models using quantization techniques (Liang et al., 2021) can be exploited to diagnose ASD in the future. Memory constraints are one of the research challenges of ASD diagnosis using MRI neuroimaging modalities. In medicine, cloud computing is one of the novel technologies to address storage and data processing issues (Chen and Ran, 2019). Using cloud computing in future work may lead to other valuable research in ASD diagnosis. In this way, MRI data is first sent to the cloud for storage. Next, the implementation of DL algorithms for the diagnosis of ASD can be carried out on their computing servers.

## Author contributions

ASh, NG, PM, DS, RA, and UA contributed to conceptualization. ASh, SL, AK, JG, and ASu contributed to methodology. SL, AK, SS-A, SA, ASu, JG, RA, and UA contributed to validation. MJ, RA, DS, PM, and SA contributed to formal analysis. ASh, SS-A, SA, MK, MJ, and PM contributed to writing—original draft preparation. ASu, SS-A, SA, and MK contributed to writing—review and editing. All authors have read and agreed to the published version of the manuscript.
