# Supplementary material for: Automatic autism spectrum disorder detection using artificial intelligence methods with MRI neuroimaging: A review
Source: Front Mol Neurosci. 2022 Oct 4;15:999605. doi: 10.3389/fnmol.2022.999605 (PMC9577321; doi:10.3389/fnmol.2022.999605)
Supplement: Supplementary file 1 [file Table_1.docx]

**Appendix A:**

Table S1: Automated diagnosis of ASD with MRI neuroimaging modalities using DL methods.

| Work | Datasets | Neuroimaging Modalities | Image Atlas + Pipeline | Details for Deep Learning Models | | | | |
| --- | --- | --- | --- | --- | --- | --- | --- | --- |
|  |  |  |  | Architecture | Layers | Optimizer | Loss Function | Classifier |
| (Li et al., 2018d) | Clinical | T-fMRI | MNI152 Atlas | 2CC3D | CNN (6) + Pooling (4) + FC (2) | -- | BCE | MV |
|  |  | Residual fMRI |  |  |  |  |  |  |
| (Li et al., 2019b) | Clinical | T-fMRI | AAL Atlas | 2CC3D | CNN (6) + Pooling (4) + FC (3) | -- | -- | Sigmoid |
| (Zhao et al., 2017b) | HCP | T-fMRI | -- | 3D-CNN | CNN (2) + LReLU + Pooling (1) + FC (1) | SGD | MNLL | Softmax |
|  |  | rs-fMRI |  |  |  |  |  |  |
| (Dvornek et al., 2018b) | Clinical | T-fMRI | AAL Atlas | LSTM | LSTM (1) + Pooling (1) + FC (3) | Adadelta | MSE | Sigmoid |
| (Leming et al., 2020) | Different Datasets | T-fMRI | AAL Atlas | 2D-CNN | CNN (2) + ReLU + BN (4) + FC (3) | Adam | -- | Softmax |
|  |  | rs-fMRI |  |  |  |  |  |  |
|  |  | Phenotypic Info |  |  |  |  |  |  |
| (Li et al., 2018e) | Clinical | T-fMRI | AAL Atlas | 2CC3D | CNN (6) + Pooling (4) + FC (2) | -- | -- | Sigmoid |
| (Li et al., 2018e) | ABIDE-I | rs-fMRI | AAL Atlas | 2CC3D | CNN (6) + Pooling (4) + FC (2) | -- | -- | Sigmoid |
| (Khosla et al., 2018) | ABIDE 1 | rs-fMRI | All Atlases + CPAC Pipeline | 3D-CNN | CNN (2) + ELU + Pooling (2) + FC (2) | SGD | -- | Sigmoid |
|  | ABIDE II |  |  |  |  |  |  |  |
| (Eslami et al., 2019) | ABIDE 1 | rs-fMRI | CC-200 and AAL Atlas + CPAC Pipeline | AE | Standard AE with Tanh Activation | -- | MSE | SLP |
|  |  |  |  |  |  |  | BCE |  |
| (Anirudh and Thiagarajan, 2019) | ABIDE 1 | rs-fMRI | HO Atlas + CPAC Pipeline | G-CNNs | Proposed G-CNN with 3 Layer CNN | Adam | -- | Softmax |
| (Brown et al., 2018) | ABIDE 1 | rs-fMRI | AAL Atlas + CCS Pipeline | BrainNet | Element-Wise (1) + E2E (2) + E2N (1) + N2G (1) + FC (3) + Leaky ReLU + Tanh | Adam | Proposed Loss function | Softmax |
| (Liao and Lu, 2018) | ABIDE 1 | rs-fMRI | AAL Atlas | DAE | Standard DAE | -- | Proposed Loss function | -- |
| (Yang et al., 2018) | ABIDE | rs-fMRI | NA | LeNet-5 | Standard LeNet-5 Architecture | -- | -- | Softmax |
| (Guo et al., 2017) | ABIDE 1 | rs-fMRI | AAL Atlas + NIAK Pipeline | SAEs | SAE with LSF Activation | LBFGS | -- | Softmax |
| (Aghdam et al., 2019) | ABIDE 1 | rs-fMRI | NA | MCNNE | CNN (3) + ReLU + Pooling (3) + FC (1) | Adam | BCE | Binary SR |
|  | ABIDE-II |  |  |  |  | Adamax |  |  |
|  | ABIDE 1 + II |  |  |  |  |  |  |  |
| (Khosla et al., 2019) | ABIDE | rs-fMRI | All Atlases + CPAC Pipeline | 3D-CNN | CNN (2) + ELU + Pooling (2) + FC (3) | SGD | BCE | Various Methods |
|  |  |  |  |  |  | Adam | MSD |  |
| (Choi, 2017) | ABIDE 1 | rs-fMRI | AAL Atlases + DPARSF Pipeline | VAE | VAE with 3 Layers | Adadelta | Proposed Loss Function | -- |
| (Dvornek et al., 2017) | ABIDE 1 | rs-fMRI | CC200 Atlases + CCS Pipeline | LSTM | LSTM (1) + Pooling (1) + FC (1) | Adadelta | BCE | Sigmoid |
| (Lu et al., 2020) | ABIDE | rs-fMRI | CC200 Atlases | SAE | SAE (3) + Sigmoid | Proposed Opt.  L-BFGS | MSE | Clustering |
|  |  | Phenotypic Info |  |  |  |  |  |  |
| (Xiao et al., 2018) | ABIDE | rs-fMRI | -- | SAE | LSTM (1) + Pooling (1) + FC (1) | Adadelta | BCE | Sigmoid |
| (Dvornek et al., 2019) | ABIDE 1 | rs-fMRI | AAL Atlases + CCS Pipeline | LSTM | LSTM (2) + Pooling (1) + FC (2) | Adam | BCE | Sigmoid |
|  |  |  |  |  |  |  | MSE |  |
| (Niu et al., 2020) | ABIDE 1 | rs-fMRI | Different Atlas | DANN | 3 MLP (1 Dropout + 4 Dense) + Self-Attention (3) + Fusion (3) + Aggregation + Dense (1) + ReLU, ELU, and Tanh | -- | SE | Sigmoid |
| (Li et al., 2018b) | ABIDE | rs-fMRI | AAL Atlas + CCS Pipeline | SSAE | 3 SSAE Layers | Gradient Descent | Proposed Loss Function | Softmax Regression |
| (El Gazzar et al., 2019) | ABIDE 1 +II | rs-fMRI | Different | 1D-CNN | CNN (1) + Pooling (1) + FC (1) | Adam | -- | Softmax |
| (Ahmed et al., 2020b) | ABIDE 1 | rs-fMRI | Different Pipelines | Various Models | CNN (6) + Pooling (4) + BN (2) + FC (2) | Adam | Propose Loss Function | Sigmoid |
| (Zhao et al., 2019) | ABIDE-II | rs-fMRI | NA | 1D-CAE | Encoder (4) + Decoder (4) + CNN (2) + pooling (2) + FC (2) | -- | -- | -- |
| (Pugazhenthi et al., 2019) | ABIDE | rs-fMRI | CCS Pipeline | AlexNet | Standard Architecture | -- | CE | Softmax |
| (Eslami et al., 2021b) | ABIDE | rs-fMRI | Different Pipelines | ASDDiagNet | Proposed DiagNet | -- | -- | SLP |
| (Eslami and Saeed, 2019) | ABIDE 1 | rs-fMRI | SCSC Atlas + CPAC Pipeline | Auto-ASD | Proposed Auto-ASD-Network | -- | NLLF | SVM |
| (Sherkatghanad et al., 2020) | ABIDE 1 | rs-fMRI | CC400 Atlas + CPAC Pipeline | 2D-CNN | CNN (7) + Pooling (7) + FC (3) | -- | -- | MLP |
| (Sairam et al., 2019) | ABIDE | rs-fMRI | NA | CNN-AE | Proposed SDAE-CNN with 7 Layes CNN | -- | -- | Softmax |
| (Dolz et al., 2018) | ABIDE 1 | rs-fMRI | NA | 3D-FCNN | CNN (9) + PReLU + FC (3) | SGD | CE | Softmax |
| (Wang et al., 2019a) | ABIDE 1 | rs-fMRI | AAL Atlas + DPARSF Pipeline | SSAE | 2 Layers SSAE | -- | -- | Softmax |
| (Zhao et al., 2018b) | ABIDE 1 | rs-fMRI | NA | 3D-CNN | CNN (7) + Pooling (3) + FC (2) + Log-Likelihood Activation | SGD | MNLL | -- |
| (Parisot et al., 2018) | ABIDE 1 | rs-fMRI | HO Atlas + CPAC Pipeline | GCN | GCN with ReLU and Sigmoid | -- | CE | Softmax |
|  |  | Phenotypic Info |  | AE | SAE with Tanh Activation |  | MSE |  |
| (Dvornek et al., 2018a) | ABIDE 1 | rs-fMRI | CC200 Atlas + CCS Pipeline | LSTM | Proposed Method | Adadelta | BCE | Sigmoid |
|  |  | Phenotypic Info |  |  |  |  | MSE |  |
| (Heinsfeld et al., 2018) | ABIDE 1 | rs-fMRI | CC200 Atlas + CPAC Pipeline | SDAE | Proposed 2-SDAE-MLP Network | -- | MSE | Softmax |
|  |  | s-MRI |  |  |  |  |  |  |
|  |  | Phenotypic Info |  |  |  |  |  |  |
| (Li et al., 2020) | Clinical | rs-fMRI | NA Atlas | 3D-CNN |  | SGD | BCE | Sigmoid |
|  |  |  |  |  | CNN (2) + ReLU + Pooling (2) + FC (2) |  |  |  |
|  |  | Fetal BOLD fMRI |  |  |  |  |  |  |
| (Akhavan Aghdam et al., 2018) | ABIDE 1 | rs-fMRI | AAL Atlas | DBN | DBN with 5 Hidden Layers | -- | -- | LR |
|  | ABIDE-II | s-MRI |  |  |  |  |  |  |
| (Mellema et al., 2019) | IMPAC | rs-fMRI | Different Atlases | Various Models | Dense (5) + LReLU | Adam | BCE | Various Methods |
|  |  | s-MRI |  |  |  |  |  |  |
| (Rakić et al., 2020) | ABIDE 1 | rs-fMRI | AAL, CC200, Destrieux Atlas + CPAC Pipeline | SAEs | 5 [ AE (3) + MLP (2)] + Softmax | -- | -- | Softmax |
|  |  | s-MRI |  |  |  |  |  |  |
| (Li et al., 2018a) | NDAR | rs-fMRI | NA | CNN | CNN (5) + ReLU + Pooling (2) + FC (5) | -- | CE | Softmax |
|  |  | s-MRI |  |  |  |  |  |  |
| (Dekhil et al., 2018) | NDAR | All Modalities | Implement the Proposed Atlas | SAE | 34 [ SAE network (2)] | PSVM | L-BFGS | Probabilistic SVM (PSVM) |
| (Li et al., 2019a) | NDAR | s-MRI | NA | DDUNET | Proposed DDUNET with 11 blocks and ReLU | SGD | CE | -- |
| (Ismail et al., 2017) | ABIDE 1 | s-MRI | NA | SNCAE | Proposed SNCAE Network | -- | -- | Softmax |
|  | NDAR/Pitt |  |  |  |  |  |  |  |
|  | NDAR/IBIS |  |  |  |  |  |  |  |
| (Kong et al., 2019) | ABIDE 1 | s-MRI | Destrieux | SpAE | SpAE with 2 Networks | -- | MSE | Softmax |
| (Pinaya et al., 2019) | HCP | s-MRI | Desikan– Killia (DDK) Atlas | DEA | AE (3) + SELU | Adam | Sum of MSE + 2 CE + CC | -- |
|  | ABIDE 1 |  |  |  |  |  |  |  |
| (Sujit et al., 2019) | ABIDE | s-MRI | NA | DCNN | CNN (6) + ReLU + Pooling (6) + FC (4) | Adam | BCE | Sigmoid |
|  | CombiRx |  |  |  |  |  |  |  |
| (Henschel et al., 2020) | ABIDE-II | s-MRI | DKT Atlas | CNN | Proposed FastSurfer CNN Network | Adam | Logistic & Dice Losses | Softmax |
| (Shahamat and Abadeh, 2020) | ABIDE 1 | s-MRI | Different | 3D-CNN | CNN (3) + ReLU + Pooling (3) + FC (2) | Adadelta | CE | Softmax |
| (Iglesias et al., 2017) | Clinical | s-MRI | NA | UNet | DCNN (7) + ReLU + Pooling (2) + BN (6) | SGD | weighted CE | Softmax |
| (Byeon et al., 2020) | ABIDE 1 | rs-fMRI | BN Atlas + CPAC Pipeline | CNN-RNN | CNN (4) + GRU (2) + ReLU + Pooling (2) + FC (5) | Adam | BCE | Sigmoid |
| (Xu et al., 2020) | Clinical | fNIRS | NA | CNN-LSTM | Proposed 1D-CNN LSTM with ReLU Activation | Adam | CCE | Bagging |
| (Xu et al., 2019) | Clinical | fNIRS | NA | CGRNN | CNN (3) + ReLU + Pooling (1) + GRU (1) + FC (1) | Adam | BCE | Sigmoid |
| (Thyreau and Taki, 2020) | Different Datasets | s-MRI | Different Atlas | CNN | Variation of the U-net Convolutional Architecture | Adam | Proposed Loss Function | -- |
| (Thomas et al., 2020) | ABIDE 1+II | rs-fMRI | HO Atlas + CPAC Pipeline | 3D-CNN | CNN (2) + ELU + Pooling (2) + FC (2) | SGD | BCE | MV |
| (El-Gazzar et al., 2019) | ABIDE 1 | rs-fMRI | CPAC Pipeline | CNN-RNN | CNN (8) + Conv-BiLSTM (2) + Sigmoid + Pooling (1) + FC (1) | Adam | CE | Softmax |
|  |  | Phenotypic Info |  |  |  |  |  |  |
| (Mostafa et al., 2019b) | ABIDE 1 | rs-fMRI | NA | AE | Proposed AE with 7 Layers | -- | -- | DNN |
|  |  | s-MRI |  |  |  |  |  |  |
| (Jiao et al., 2020) | ABIDE 1 | rs-fMRI | CC200 Atlas + CPAC Pipeline | CapsNet | Standard Architecture | Adam | Proposed Loss Function | K-Means Clustering |
| (Bengs et al., 2020) | ABIDE | rs-fMRI | CCS Pipeline | convGRU-CNN3D | convGRU+ 3D CNN | Adam | CE | -- |
| (Gupta et al.) | ABIDE | rs-fMRI | CC200 Atlas + CPAC Atlas | 1D-CNN | Conv (3) + Pooling (3) + FC (1) | -- | -- | Softmax |
| (Wang et al., 2020b) | ABIDE | rs-fMRI | CC200, AAL, Dosenbach Atlas + CPAC Pipeline | SDA | 3 DAEs (Each 3 Hidden Layers) | -- | -- | Different Classifier |
| (Tang et al., 2020) | ABIDE 1 | rs-fMRI | AAL Atlas + CPAC Pipeline | Proposed CNN Method | Conv (1) + Max (1) + Res-blocks (4) + Average Pooling (1) + 1 FC + ReLU | Mini-Batch SGD | Softmax CE | 4 FC |
| (Ke et al., 2020) | ABIDE | MRI | NA | DNN | Different Configurations | -- | -- | -- |
| (Ahmed et al., 2020a) | ABIDE | rs-fMRI | DPARSF Pipeline | RBM | Proposed Architecture | -- | -- | SVM |
| (Ronicko et al., 2020) | ABIDE 1 | rs-fMRI | GCA Atlas | 1D-CNN | Conv (3) + Pooling (1) + FC (2) + ReLU  Conv (3) + BN (3) + Pooling (4) + FC (1) + ReLU | -- | CE | Softmax |
|  | ABIDE II |  | HO Atlas |  |  |  |  |  |
|  |  |  | DCA Atlas |  |  |  |  |  |
| (Zhang et al., 2020b) | IMPAC | sMRI | Proposed Atlas | 2D-CNN | Conv (3) + Pooling (1) + FC (2) + ReLU  Conv (3) + BN (3) + Pooling (4) + FC (1) + ReLU | Proposed Opt | -- | Softmax |
|  |  | rs-fMRI |  |  |  |  |  |  |
| (Shahamat and Abadeh, 2020) | ABIDE 1 | MRI | HO, SSA Atlas | 3D-CNN | Conv (3) + Pooling (3) + FC (2) + ReLU | Adadelta | CE | Softmax |
| (Felouat and Oukid-Khouas, 2020) | ABIDE 1 | rs-fMRI | CC400 Atlas + DPARSF Pipeline | GCN | Proposed Architecture | -- | -- | Majority Voting |
| (Geng et al., 2020) | ABIDE | fMRI | CC200 Atlas | GAN | Encoder, Generator, Discriminator Networks | Adam | -- | 3-Layer DNN |
| (Al-Hiyali et al., 2021) | ABIDE | rs-fMRI | AAL Atlas | PreTrained Artcitectures | Densenet201, GoogleNet, Resnet101, Resnet18 | -- | -- | SVM, KNN |
| (Tummala, 2021) | ABIDE 1 | sMRI | NA | ResNet50 | Standard Architecture | Adam | -- | Sigmoid |
| (Pominova et al., 2021) | ABIDE II | rs-fMRI | AAL Atlas | Proposed 3D CNN Method | 3D Convolutional Autoencoders | -- | -- | SVM |
| (Yin et al., 2021) | ABIDE 1 | rs-fMRI, MRI | Various Atlas | CNN-AE | Hidden Layers (4) | Adam | CE | Softmax |
| (Hiremath et al., 2020) | ABIDE 1 | sMRI | DK Atlas | DenseNet | 4 Dense Blocks | -- | -- | LDA |
|  | ABIDE II |  |  |  |  |  |  |  |
| (Du et al., 2020) | ABIDE 1 | fMRI, sMRI | NA | 2D-CNN and 3D-CNN | Conv (3) + Pooling (3) + FC (2) | -- | -- | Different Methods |
| (Haweel et al., 2021b) | NDAR | Task fMRI | HO Atlas | 2D CNN | Conv (3) + Pooling (3) + FC (2) | SGD | -- | Softmax |
| (Wang et al., 2021a) | ABIDE | rs-fMRI | CC200 Atlas + CCS Pipeline | cGCN | Conv (5) + RNN (Temporal Average Pooling Layer) | Adam | -- | Softmax |
| (Gupta et al.) | ABIDE | rs-fMRI | CC200 Atlas + CPAC Pipeline | 1D-CNN | Conv (3) + Pooling (3) + FC (1) | -- | -- | Softmax |
| (Yang et al., 2020) | ABIDE 1 | rs-fMRI | CC400 Atlas + CPAC Pipeline | DNN | Hidden Layers (2) | Adam | BCE | Softmax |
| (Hu et al., 2020) | ABIDE 1 | rs-fMRI | AAL Atlas + CPAC Pipeline | FCNN | FC (2) + BN (2) + LeakyReLu | Adam | BCE | Sigmoid |
| (Mahmoud et al., 2020a) | ABIDE 1 | rs-fMRI | CC200 Atlas + CPAC Pipeline | DCAE | Encoder: 4 CNN Networks [Conv (4) + BN (3) + Pooling (2) + ReLU], Decoder: 4 CNN Networks [Reverse Configuration] | -- | -- | FC Layer + Dense Layer |
| (Shrivastava et al., 2020) | ABIDE 1 | rs-fMRI | CC400 Atlas + CPAC Pipeline | 2D-CNN | Conv (2) + EvoNorm-S0 (2) + Tanh + FC Layer (1) | Adam | BCE | Sigmoid |
| (Gao et al., 2021) | ABIDE 1 | sMRI | SRI24 Atlas | ResNet | Conv Blocks (5) | Adam | BCE | FC Network |
| (Sewani and Kashef, 2020) | ABIDE 1 | rs-fMRI | CPAC Pipeline | CNN-AE | AE Network + 1D-Conv (2) + BN (2) + ReLU + Pooling (2) + FC (2) |  |  | Sigmoid |
| (You et al., 2020) | ABIDE | fMRI | AAL Atlas | FC | Pooling (2) +Conv (3) + FC (1) + ReLU | Adam | -- | Softmax |
|  |  |  |  | ALFF Net | Pooling (2) + 3D-Conv (4) + FC (1) + ReLU |  |  |  |
| (Ji et al., 2021) | ABIDE 1 | ??? | DK Atlas | CGTS-GAN | Proposed Architecture | -- | -- | -- |
| (Sserwadda and Rekik, 2021) | ABIDE 1 | Rs-fMRI | AAL Atlas + DPARSF Pipeline | CNN | NA | -- | -- | Softmax |
|  | ABIDE II |  |  |  |  |  |  |  |
| (Mahmoud et al., 2020b) | ABIDE 1 | rs-fMRI | CPAC Pipeline | SAE | Two unsupervised Sparse AEs | -- | -- | Supervised AE |
| (Bayram et al., 2021) | ABIDE 1 | rs-fMRI | BASC Pipeline | CNN-RNN | Different Architectures | -- | -- | Sigmoid |
| (Mozhdefarahbakhsh et al., 2021) | ABIDE 1 | rs-fMRI | MSDL Pipeline | 1D-CNN | Conv (4) + BN (3) + Pooling (3) + FC (2) + ReLU | -- | -- | Sigmoid |
| (Dominic et al., 2021) | ABIDE 1 | rs-fMRI | CCS Pipeline | Inception-ResNetV2 | InceptionResNetV2 Architecture + Pooling Layers | SGD | -- | Softmax |
| (Husna et al., 2021) | ABIDE 1 | rs-fMRI | NA | ResNet-50 | Standard Architecture | -- | -- | Softmax |
|  | ABIDE II |  |  |  |  |  |  |  |
| (Almuqhim and Saeed, 2021) | ABIDE 1 | rs-fMRI | CC200 + CPAC Pipeline | ASD-SAENet | SAE + DNN | Adam | CE | Softmax |
| (Subah et al., 2021) | ABIDE 1 | rs-fMRI | BASC, CC200, and AAL Atlas + CPAC Pipeline | DNN | Hidden (2) + ReLU | -- | -- | Sigmoid |
| (Huang et al., 2020b) | ABIDE 1 | rs-fMRI | CC200 Atlas + CPAC Pipeline | DBN | 3 RBM Layers | RMSProp | CE | Softmax |
| (Leming et al., 2021) | Different Dataset | sMRI and fMRI | AAL Atlas | 2D-CNN | Conv (1) + FC (3) + BN (3)+ ReLU | Adam | -- | Softmax |
| (Ji and Yao, 2020) | ABIDE 1 | rs-fMRI | AAL Atlas + DPARSF Pipeline | CNNGLasso | Conv Layer + FC Layer | Adam | Proposed Loss Function | Softmax |
| (Arya et al., 2020) | ABIDE 1 | sMRI, fMRI | Various Atlas | GCN | p-GCN + s-GCN + ss-GCN Networks | -- | -- | -- |
|  | ABIDE II |  |  |  |  |  |  |  |
| (Dsouza et al., 2021) | KKI | rs-fMRI, DTI | AAL Atlas | MGCN | Proposed Architecture | SGD | Combined Loss Function | ANN |
|  | HCP |  |  |  |  |  |  |  |
| (D’Souza et al., 2020) | Clinical | rs-fMRI, DTI | AAL Atlas | LSTM-ANN | LSTM (2) + Both the P-ANN and the A-ANN Have 2 Hidden Layers + ReLU | Adam | Proposed Loss Function | Attention-Weighted Average |
| (Haweel et al., 2021a) | NDAR | T-fMRI, sMRI | HO | 1D-CNN | CNN (2) + ReLU + Pooling (2) + FC (1) | SGD | -- | Softmax |
| (Jönemo et al., 2021) | ABIDE | rs-fMRI | CCS | 3D-CNN | CNN (3) + ReLU + Pooling (3) + FC (1) | Adam | -- | Sigmoid |
| (Wang et al., 2022) | ABIDE | rs-fMRI | 6 Atlases + CPAC | Multi-Atlas Graph Convolutional Network Method (MAGCN) | GCN Model | -- | Cross Entropy | Stacking Ensemble Learning Method + Ridge Classifier |
| (Jha et al., 2021) | ABIDE I | rs-fMRI | -- | MHATC | Multi-Head Attention Encoder (MHAE) + Temporal Consolidation Module (TCM) | -- | Cross Entropy | Pooling (1) + FC (1) |
| (Zhang et al., 2022) | ABIDE I | rs-fMRI | CC400 + CPAC | Simplified VAE Unsupervised Pretraining And MLP Supervised Fine-Tuning | Hidden Layers (3) | RMSProp | Cross Entropy | Softmax |
| (Dekhil et al., 2017) | NDAR | sMRI, fMRI | Talairach | Deep Fusion Classification Network (DFCN) | 2 Stacked Autoencoder With Non-Negativity Constraint (SNCAE) | -- | -- | Softmax |
| (Yang et al., 2021) | ABIDE | sMRI | -- | 2D CAM, 3D CAM, 3D Grad-CAM | Proposed Architectures | -- | Cross Entropy | Classification Output Layer |
| (Chen et al., 2021b) | ABIDE | rs-fMRI | HO + CPAC | Invertible Dynamic GCN (ID-GCN) | Three Invertible Blocks (2 Different GCN) | -- | Cross Entropy | Softmax |
| (Li et al., 2021) | ABIDE | rs-fMRI | AAL, HO, MODL | Functional Graph Discriminative Network (FGDN) | Functional Graph Construction Layer (1) + Graph Conv Layers (2) + PReLU + FC Layer (1) | Adam | Proposed Loss Function | Sigmoid |
| (Shao et al., 2021) | ABIDE | rs-fMRI, Phenotypic | CPAC + HO | Combined DFS and GCN Method | Sparse One-To-One Linear Layer + Hidden Layers (3) + Graph Conv Layers + ReLU + Dropout Layer | Adam | Proposed Loss Function | Softmax |
| (Chen et al., 2021a) | ABIDE | rs-fMRI, Phenotypic | -- | Adaptive Multi-Layer Aggregation Graph Convolutional Network (AMA-GCN) | Proposed Architectures | Adam | Fusion Loss | Softmax |
| (Cao et al., 2021) | ABIDE I | rs-fMRI, Phenotypic | CPAC + HO | DeepGCN | 16 Layers GCN + Dropout + ResNet Units + DropEdge Strategy | -- | -- | Softmax |
| (Chu et al., 2022) | ABIDE | rs-fMRI | AAL, CC200 + DPARSF | Multi-Scale Graph Representation Learning (MGRL) Framework | Multi-Scale FCNs Construction + FCNs Representation Learning Via Multi-Scale GCNs + Multi-Scale Feature Fusion and Classification | Adam | Cross Entropy | Softmax |
| (Last access 19/07/2022c) | ABIDE | rs-fMRI | -- | CNN | CNN (3) + ReLU + Pooling (2) + FC (2) | -- | -- | Softmax |
| (Yin et al., 2022) | ABIDE I | rs-fMRI | -- | SSAE + MLP | Dense Layers (4) + ReLU + Dense Layers (4) | -- | Cross Entropy | Softmax |
| (Wen et al., 2022) | ABIDE | rs-fMRI | AAL, CC200 + CPAC | Multi-View Graph Convolutional Neural Network (MVS-GCN) | Graph Structure Learning (GSL) + Multi-Task Graph Embedding Learning for Different Views of Brain Networks (MVL) + View Consistency Regularization (VCR) and the Prior Subnetwork Structure Regularization (SNR). | Adam | Proposed Loss Function | -- |
